# 7-oxo-DHEA enhances impaired *M. tuberculosis*-specific T cell responses during HIV-TB coinfection

**DOI:** 10.1186/s12929-019-0604-z

**Published:** 2020-01-06

**Authors:** María Belén Vecchione, Natalia Laufer, Omar Sued, Marcelo Corti, Horacio Salomon, Maria Florencia Quiroga

**Affiliations:** 1 Consejo Nacional de Investigaciones Científicas y Técnicas (CONICET), Universidad de Buenos Aires, Instituto de Investigaciones Biomédicas en Retrovirus y Sida (INBIRS), Buenos Aires, Argentina; 2 Área de Investigaciones Clínicas, Fundación Huésped, Buenos Aires, Argentina; 3 División “B” VIH/Sida, Hospital Francisco J. Muñiz, Buenos Aires, Argentina

**Keywords:** Human, Infectious immunity-bacteria, Hormone modulatory mechanisms, Cytokines, Effector T cells

## Abstract

**Background:**

*Mycobacterium tuberculosis* (*Mtb*) is the causative agent of tuberculosis (TB), affecting approximately one third of the world’s population. Development of an adequate immune response will determine disease progression or progress to chronic infection. Risk of developing TB among human immunodeficiency virus (HIV)-coinfected patients (HIV-TB) is 20–30 times higher than those without HIV infection, and a synergistic interplay between these two pathogens accelerates the decline in immunological functions. TB treatment in HIV-TB coinfected persons is challenging and it has a prolonged duration, mainly due to the immune system failure to provide an adequate support for the therapy. Therefore, we aimed to study the role of the hormone 7-oxo-dehydroepiandrosterone (7-OD) as a modulator of anti-tuberculosis immune responses in the context of HIV-TB coinfection.

**Methods:**

A cross-sectional study was conducted among HIV-TB patients and healthy donors (HD). We characterized the ex vivo phenotype of CD4 + T cells and also evaluated in vitro antigen-specific responses by *Mtb* stimulation of peripheral blood mononuclear cells (PBMCs) in the presence or absence of 7-OD. We assessed lymphoproliferative activity, cytokine production and master transcription factor profiles.

**Results:**

Our results show that HIV-TB patients were not able to generate successful anti-tubercular responses in vitro compared to HD, as reduced IFN-γ/IL-10 and IFN-γ/IL-17A ratios were observed. Interestingly, treatment with 7-OD enhanced Th1 responses by increasing *Mtb*-induced proliferation and the production of IFN-γ and TNF-α over IL-10 levels. Additionally, in vitro *Mtb* stimulation augmented the frequency of cells with a regulatory phenotype, while 7-OD reduced the proportion of these subsets and induced an increase in CD4 + T-bet+ (Th1) subpopulation, which is associated with clinical data linked to an improved disease outcome.

**Conclusions:**

We conclude that 7-OD modifies the cytokine balance and the phenotype of CD4 + T cells towards a more favorable profile for mycobacteria control. These results provide new data to delineate novel treatment approaches as co-adjuvant for the treatment of TB.

## Background

HIV and *Mycobacterium tuberculosis* (HIV-TB) coinfection represents a challenge for the study of its physiology, since the presence of both pathogens is characterized by persistent immune dysregulation and altered cytokine profile. Although highly active antiretroviral therapy impedes HIV replication and leads to increased CD4 + T cell numbers, *Mtb*-specific responses do not change substantially over the first six months on antiretroviral therapy [1]. This situation unravels the need for developing additional immunotherapies to protect from and control *Mtb* infection, especially in HIV+ individuals. The identification of host factors that promote disease progression or control may lead to the discovery of new host-directed therapies (HDT). In the context of HIV-TB coinfection, these treatments should aim to enhance antigen-specific immune responses, reduce excess inflammation, preserve cell function or improve the effectiveness of conventional therapies. HDT could offer additional advantages for coinfected patients since they may reduce the length of treatments, achieving better outcomes and/or decreasing the chances of relapse or reinfection [2, 3].

Different cell subpopulations are involved in active protection against *M. tuberculosis* (*Mtb*) infection. In particular, CD4 + T cells restrict bacterial growth through the production of cytokines that activate macrophages [4]. The variability in the quality and quantity of cytokines produced at the onset of T cell activation depends on the nature of the threat encountered [5]. IFN-γ mediates macrophage activation through the induction of phagocytosis, autophagy and antigenic presentation, and contributes to the resistance and eradication of intracellular pathogens [6, 7]. Additionally, TNF-α is essential for the prevention of *Mtb* infection and maintenance of latent TB infection [8, 9]. In contrast, IL-10 is a regulatory cytokine that protects the host from excessive inflammation and tissue damage and also inhibits immune responses [10, 11]. Lastly, IL-17A contributes both to the protection and the pathology of TB because it is involved in the formation of mature granuloma [12], but also it mediates the recruitment of neutrophils, which are related to pathological damage of the lung [13].

To date, few studies have explored the effects of immunomodulatory compounds on the function of T cell effectors in the context of TB, particularly during HIV coinfection [14]. Our research group has published several data on this subject, since we have studied the role of DHEA in the context of HIV-TB coinfection for years [15, 16]. In a recent report, we demonstrated the presence of a hormonal imbalance in HIV-TB patients, who exhibited higher plasma levels of DHEA and its androstenetriol (AET) and 7-oxo-DHEA (7-OD) metabolites. Remarkably, we found that higher concentrations of 7-OD positively correlated with absolute CD4 + T cell counts and nadir CD4 + T cell values and also was associated to lung-restricted TB infection [17].

Given the importance of CD4 + T cells over the control of mycobacterial growth and the reported effect of DHEA as an immune-modulating hormone, this work aimed to study the effect of 7-OD on *Mtb*-induced cell proliferation, cytokine production and master transcription factors expression in CD4 + T cells. Our results showed that *Mtb* induced a response with a regulatory phenotype instead of an effector response. On the contrary, 7-OD modified the effect caused by *Mtb*-stimulation, inducing a Th1 type response by increasing lymphoproliferation, the production of IFN-γ and TNF-α over IL-10 levels and induced an augment in CD4+ T-bet+ (i.e., Th1) subpopulation. Therefore, we conclude that 7-OD modifies the balance of cytokines and the profile of CD4 + T cells towards a more favorable profile for mycobacteria control. These results provide new data to delineate novel HDT approaches as co-adjuvants for the treatment of TB.

## Methods

### Study population

This is a cross-sectional study. A total of 62 subjects (Table 1) were recruited and classified into two groups: 1) chronic HIV-1+ infected individuals with active TB (HIV-TB), who received none or less than two weeks of anti-TB therapy at the moment of sample collection; 2) healthy donors (HD), with no history of TB, HIV or systemic infections. HIV-TB patients were evaluated at the Hospital de Infecciosas “Dr. Francisco Javier Muñiz”, Buenos Aires, Argentina. HIV and TB diagnosis was determined following current guidelines. Some individuals were on anti-retroviral treatment [18]. Peripheral blood buffy-coats from HD were kindly provided by the Hemotherapy Division of Sanatorio “Dr. Julio Mendez”, Buenos Aires, Argentina. None of the subjects had metabolic or endocrine disorders or received DHEA or glucocorticoids. The entire group of individuals had been BCG (Bacillus Calmette-Guerin)-vaccinated at birth.

**Table 1:** Characteristics of the subjects enrolled in the study. IQR: interquartile range. Mann-Whitney U test, **p < 0.01 and ****p < 0.001

Characteristic	HIV-TB (*n* = 34)	HD (*n* = 28)
Median CD4 + T cell count (cell/ml)	162.5 (IQR: 34.8–270.0)	789.0 (IQR: 627.2–1031.0) ****
Median viral load (copies/ml)	86,637 (IQR: 9536–241,280)	N/A
Median DHEA plasma concentration (ng/ml)	4.4 (IQR: 2.3–8.7)	0.8 (IQR: 0.7–4.4) **
Median 7-OD plasma concentration (ng/ml)	10.9 (IQR: 4.7–45.7)	5.0 (IQR: 3.0–7.1) **

### Antigens, mitogens and steroids

Pre-titrated *Mycobacterium tuberculosis* (*Mtb*) H37Rv gamma-irradiated whole cells were obtained from BEI Resources, NIAID, NIH (NR-14819); Phytohemmaglutinin (PHA) and DHEA were acquired from Sigma-Aldrich, and 7-OD was purchased from Steraloids Inc.

### Culture conditions

EDTA-anticoagulated blood samples were drawn during morning hours. PBMCs were isolated by density gradient centrifugation on Fycoll-Paque® and cultured in RPMI medium (Sigma-Aldrich) supplemented with 10% fetal bovine serum (PAA), 2mML-glutamine (Gibco BRL), 100 U/mL penicillin (PAA) and 100 mg/mL streptomycin (Gibco BRL) at 37 °C in a humidified CO_2_ containing atmosphere. PBMCs were stimulated with *Mtb* H37Rv gamma-irradiated whole cells (10 μg/mL) and treated with DHEA or 7-OD (at 1 × 10^−9^M, 1 × 10^−8^M, 1 × 10^−7^M and 1 × 10^−6^M). We chose steroid concentrations that encompass the spectra ranging from sub-physiological to pharmacological levels, according to our previous work [17].

### Cell proliferation assay and cytokine production

PBMCs were plated at 2.5 × 10^5^ cells/well in a U-bottom 96-well plate, as described above. Specific lymphoproliferation was assessed by measuring [methyl-^3^H] thymidine (GE Healthcare) incorporation. PBMCs were cultured for 96 h, when [methyl-^3^H] thymidine (0.5 μCi per well) was added and incubated for another 24-h period. Cells were harvested by pouring them onto glassfiber filters (Whatman GF/A). Filters were placed into vials containing liquid scintillation cocktail (2 ml/disc, Optiphase Hisafe 2, PerkinElmer) to determine radiation (counts per minute, CPM) in a liquid scintillation counter (LKB Wallac 1214 RackBeta). To assay cytokine production, cell-free culture medium was obtained from cultures assayed at 120 h. Supernatants were centrifuged for 3 min at 13000 rpm, collected and stored at − 20 °C until cytokine measurement was performed by ELISA, according to the manufacturer’s instructions. IFN-γ, IL-10, TNF-α (BD OptEIA™) and IL-17A (BioLegend) were quantified.

### Flow cytometry

Ex-vivo and in-vitro analysis were conducted. Recently thawed or freshly isolated PBMCs were stained with fluorochrome-conjugated antibodies (Abs) for ex vivo study: PE-Cy7 anti-CD3, BV510 anti-CD4, FITC anti-CD25, PE anti-FoxP3, Alexa Fluor 647 anti-ROR-γt, (BD Biosciences) and PerCP/Cy5.5 anti-T-bet (BioLegend). For the in vitro tests, PBMCs were first plated at 1 × 10^6^ cells/ml in a 48-well plate, and incubated as described above. Cells were treated with 7-OD at 1 × 10^−6^M or DHEA at 1 × 10^−7^M. After 72 h, cells were harvested, stained, and analyzed by flow cytometry. FoxP3 staining protocol was used according to the manufacturer’s instructions (BD Biosciences). Negative control samples were incubated with irrelevant isotype-matched mAbs in parallel with experimental samples. Samples acquisition and analysis were carried out on a FACSCanto flow cytometer using the BD FACSDiva software (BD Biosciences). Data were analyzed with FlowJo Software (Version 10.4, Tree Star) after gating on the lymphocyte population in the FSC/SSC window and excluding cell aggregates (doublets) by FSC-A/FSC-H and FSC-H/FSC-W. Dead cells were excluded using Live/Dead viability probe (Life Technologies). Gating strategy is shown in Additional file 1: Figure S1. Boolean gating was used to define individual non-overlapping functional subsets or CD4 + T cells that expressed only two transcription factors. Frequencies were normalized to unstimulated control. Mean fluorescence intensity (MFI) was depicted as the ratio of the geometric mean MFI of the marker of interest over the MFI of the corresponding negative population. MFI is expressed as median ± interquartile range (IQR).

### Statistical analyses

Statistical analyses were conducted using GraphPad Prism 7 (GraphPad software Inc.). D’Agostino & Pearson normality test was used to assess normal distribution. Comparisons of the two groups for paired samples were assessed by the paired t test or Wilcoxon matched-pairs signed rank test, as appropriate. Alternatively, unpaired t test or Mann-Whitney U test were used to evaluate unpaired samples. Comparisons of three or more variables were done using the Friedman test, followed by post-hoc comparisons: Fisher’s LSD or Dunn’s test. Correlation analyses were determined using the Spearman’s rank test. *p* values < 0.05 were considered significant.

## Results

### Description of study cohorts

**Fig. 1:**
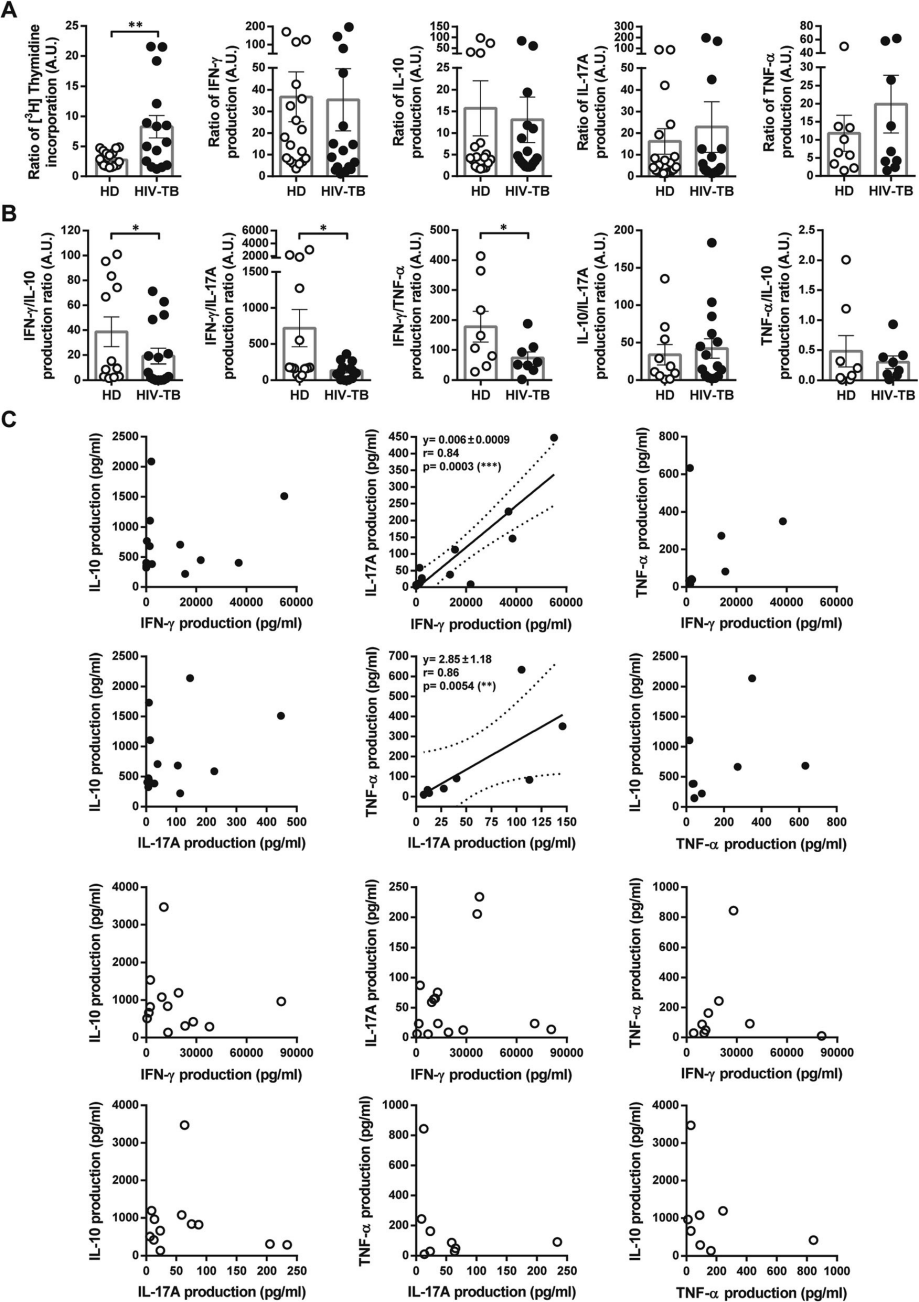
In vitro responses to *M. tuberculosis* of PBMCs from HIV-TB and HD individuals. PBMCs from HD and HIV-TB individuals were stimulated with gamma-irradiated *Mtb* (10 μg/ml) for 5 days. Each graph depicts values from HIV-TB patients (black circles) and HD (open circles). Each symbol represents an individual subject. **a** *Mtb*-induced proliferation and cytokine secretion. The Y axis shows cell proliferation or cytokine production. Values are relativized to unstimulated cells. **b** Cytokine production profiles from HD and HIV-TB in response to *Mtb*. The Y axis indicates cytokine ratio. Error bars show mean ± SEM. Unpaired t test or Mann-Whitney U test, as appropriated *p < 0.05, **p < 0.01. **c** Correlation analysis among secretion of IFN-γ, IL-10, IL-17A and TNF-α from HIV-TB and HD groups. Continuous lines represent linear regression curves, and dashed lines represent 95% confidence intervals. Spearman’s rank correlation coefficient (r), p value and slope are indicated

We initially analyzed memory responses to *Mtb* on PBMCs, studying cell proliferation and cytokine production (IFN-γ, IL-10, IL-17A and TNF-α). Cytokine production ratio and correlation analyses were also performed (Fig. 1). We observed that *Mtb*-induced lymphoproliferation in HIV-TB was higher than in HD (Fig. 1a). However, compared to HIV-TB individuals, HD produced increased levels of IFN-γ with respect to those of IL-10, IL-17A and TNF-α (Fig. 1b), suggesting a predominant Th1-type response. Alternatively, a positive association between the secretion of IL-17A with IFN-γ and TNF-α was observed in HIV-TB individuals (Fig. 1c). These data suggest that HIV-TB patients are not able to generate an adequate response for the containment of the bacteria, as a result of the reduction in IFN-γ/IL-10 and IFN-γ/IL-17A ratios and the combined production of pro-inflammatory cytokines (TNF-α and IL-17A) which unrestrained levels are associated with immunopathology [19, 20].

### 7-oxo-DHEA boosts *Mtb*-specific lymphoproliferation

**Fig. 2:**
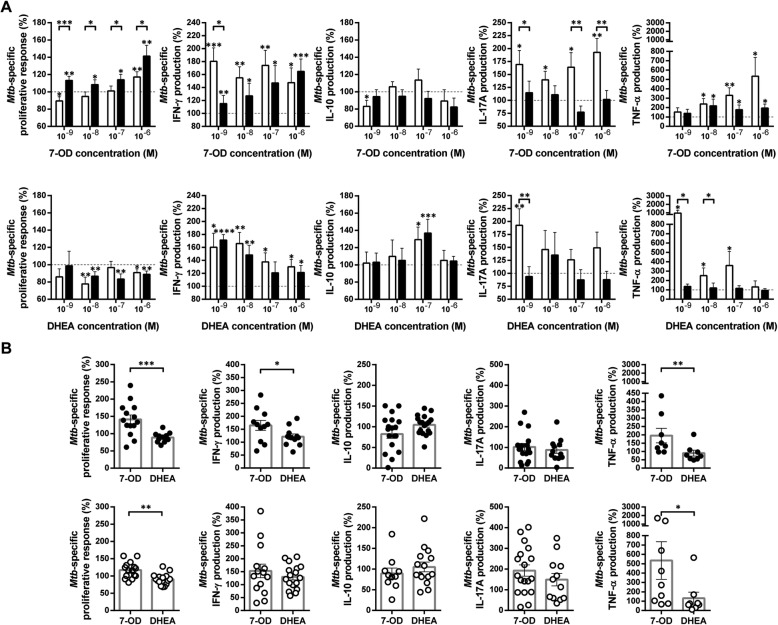
7-OD and DHEA modulate *Mtb*-specific immune responses. PBMCs from HIV-TB and HD were stimulated with gamma-irradiated *Mtb* as indicated in methods. **a** Effect of 7-OD and DHEA at different concentrations on *Mtb*-specific responses. The X axis indicates hormone concentrations. The Y axis shows cell proliferation or cytokine production. Values are relativized to cells stimulated with *Mtb* (100%). The results are plotted for HD (open bars) and HIV-TB patients (black bars). Error bars show mean ± SEM. Statistical analyses were performed using absolute values. Unpaired *t* test or Mann-Whitney U test *p < 0.05, **p < 0.01, ***p < 0.005 and ****p < 0.001. Numbers of individuals per group were from 8 to 18, according to sample availability. **b** Comparison of the immunomodulatory capacity of 7-OD and DHEA at 1 × 10^−6^M on PBMCs stimulated with *Mtb*. Values are relativized to the cells stimulated only with antigen (100%). The results are plotted for HIV-TB patients (black circles) and HD (open circles). Each symbol represents an individual subject. The statistical analysis was performed using absolute values. Unpaired t test or Mann-Whitney U test, as appropriated *p < 0.05, **p < 0.01, and ***p < 0.005. **a** and **b** To estimate the immunomodulatory effect of steroids, the response of *Mtb*-stimulated cells was defined as 100%. The calculation was: Modulation (%) = (*Mtb*-stimulated and hormone treated - unstimulated) / (*Mtb*-stimulated - unstimulated) × 100

It is generally accepted that pathogen-specific T cells exhaustion prevents lymphoproliferation and the ability to mediate effector functions during chronic infections [21]. In effect, the proliferative capacity of HIV-specific CD4 + T cells is impaired in individuals with higher viral load [22]. However, *Mtb*-specific CD4+ T cells from both of both HIV-infected and uninfected individuals, preserve a robust proliferative response [23]. Therefore, we studied the effect of 7-OD and DHEA on *Mtb*-induced cell proliferation. We observed that 7-OD significantly promoted proliferative activity*,* with a differential outcome on cells from HIV-TB individuals when compared with HD (Fig. 2a, left upper panel). Otherwise, DHEA induced anti-proliferative activity, in both HIV-TB individuals and HD (Fig. 2a, left lower panel). We contrasted the modulatory effect of 7-OD and DHEA at the highest dose (1 × 10^−6^M) and found that 7-OD significantly promotes *Mtb*-specific cell proliferation (Fig. 2b).

### 7-OD modifies *Mtb*-induced cytokine profile to a Th1-type

We aimed to study the effect of 7-OD over the cytokine pattern (IFN-γ, IL-10, IL-17A and TNF-α) secreted by *Mtb*-stimulated PBMCs. The results showed that 7-OD significantly stimulated the production of IFN-γ and TNF-α in both study groups (Fig. 2a, upper panel). No modulation by 7-OD on IL-10 and IL-17A secretion was observed in the HIV-TB group, while this hormone downregulated the production of IL-10 (1 × 10^− 9^ M) and stimulated the secretion of IL-17A in HD (Fig. 2a, upper panel). On the other hand, DHEA increased the production of IFN-γ and IL-10 in HD and HIV-TB (1 × 10^− 7^ M) (Fig. 2a, lower panel). DHEA also enhanced IL-17A and TNF-α levels in HD at low doses (Fig. 2a, lower panel), coinciding with its physiological plasma concentration [17]. 7-OD (1 × 10^−6^M) was more effective than DHEA in increasing the secretion of IFN-γ in HIV-TB and TNF-α in both groups. In contrast, no differences were observed between both hormone effects on the production of IL-10 or IL-17A (Fig. 2b).

**Fig. 3:**
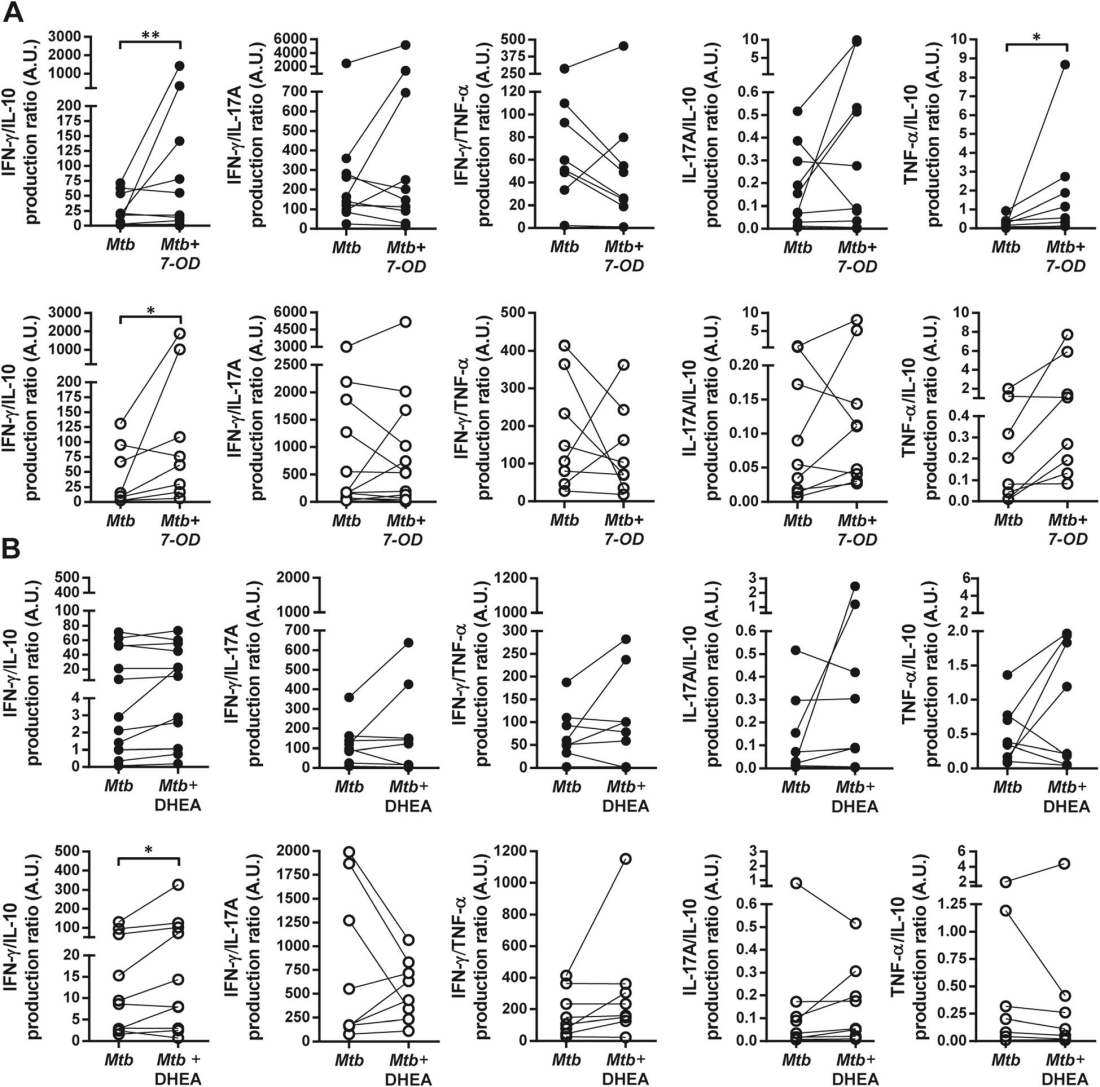
Ratios of in vitro *Mtb*-specific cytokine production are modified by 7-OD and DHEA. PBMCs from HIV-TB individuals (black circles) and HD (open circles) were stimulated with gamma-irradiated *Mtb* in the presence/absence of hormones, as specified in methods. The Y axis indicates cytokine production ratio in response to *Mtb* and its modulation by **(a)** 7-OD or **(b)** DHEA, both at 1 × 10^−6^M. Each symbol represents an individual subject. The statistical analysis was performed using absolute values. Paired t test or Wilcoxon matched-pairs signed rank test, as appropriated *p < 0.05

As *Mtb* modulate the production of cytokines in favor of a regulatory response, an important feature of an adjuvant compound is the ability to promote a Th1 profile. Thus, *Mtb*-induced cytokine ratio was analyzed. Remarkably, 7-OD (1 × 10^−6^M) managed to significantly upregulate IFN-γ/IL-10 and TNF-α/IL-10 ratios only in patients (Fig. 3, upper panel). This effect was not observed with DHEA (Fig. 3, lower panel), but both hormones induced an increment of IFN-γ/IL-10 ratio in HD. These data indicate that 7-OD was able to modulate *Mtb*-specific cell proliferation and a Th1 type cytokine profile during HIV-TB co-infection, by increasing the levels of IFN-γ and TNF-α, without suppressing the necessary action of IL-10 and IL-17A, which would mediate an optimal resistance to the mycobacteria [24].

### Characterization of CD4 + Th subpopulations in HIV-TB and HD individuals

**Fig. 4:**
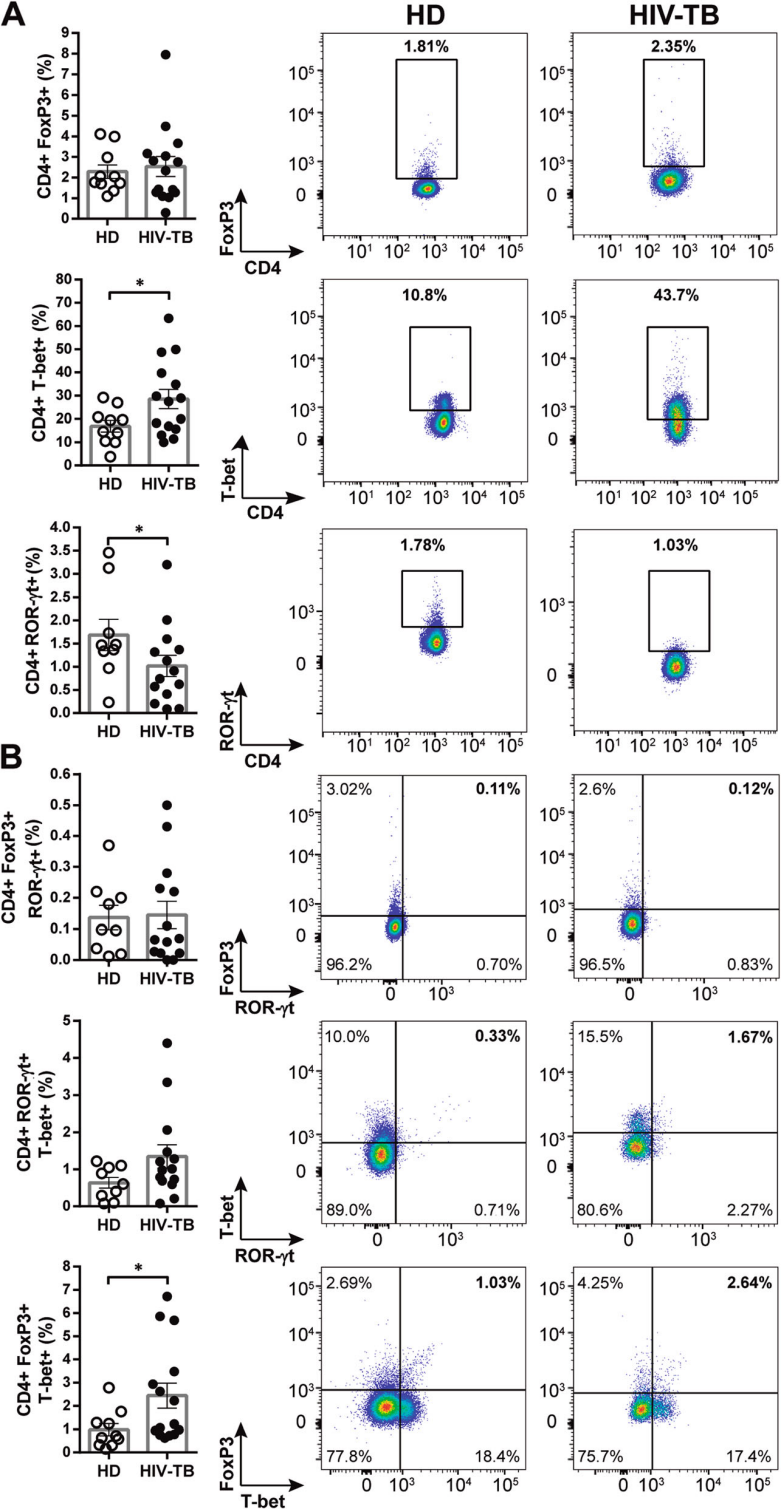
Phenotype of CD4 + T cells from HD and HIV-TB. Recently thawed or freshly isolated PBMCs were stained and analyzed by flow cytometry. Figure shows the percentage of CD4+ cells that express (**a**) FoxP3, T-bet or ROR-γt and (**b**) the co-expression of transcription factors using a Boolean gating strategy. Representative flow cytometry examples are shown. The results are plotted for HD (open circles) and HIV-TB patients (black circles). Each symbol represents an individual subject. Unpaired t test or Mann-Whitney U test, as appropriated *p < 0.05

**Fig. 5:**
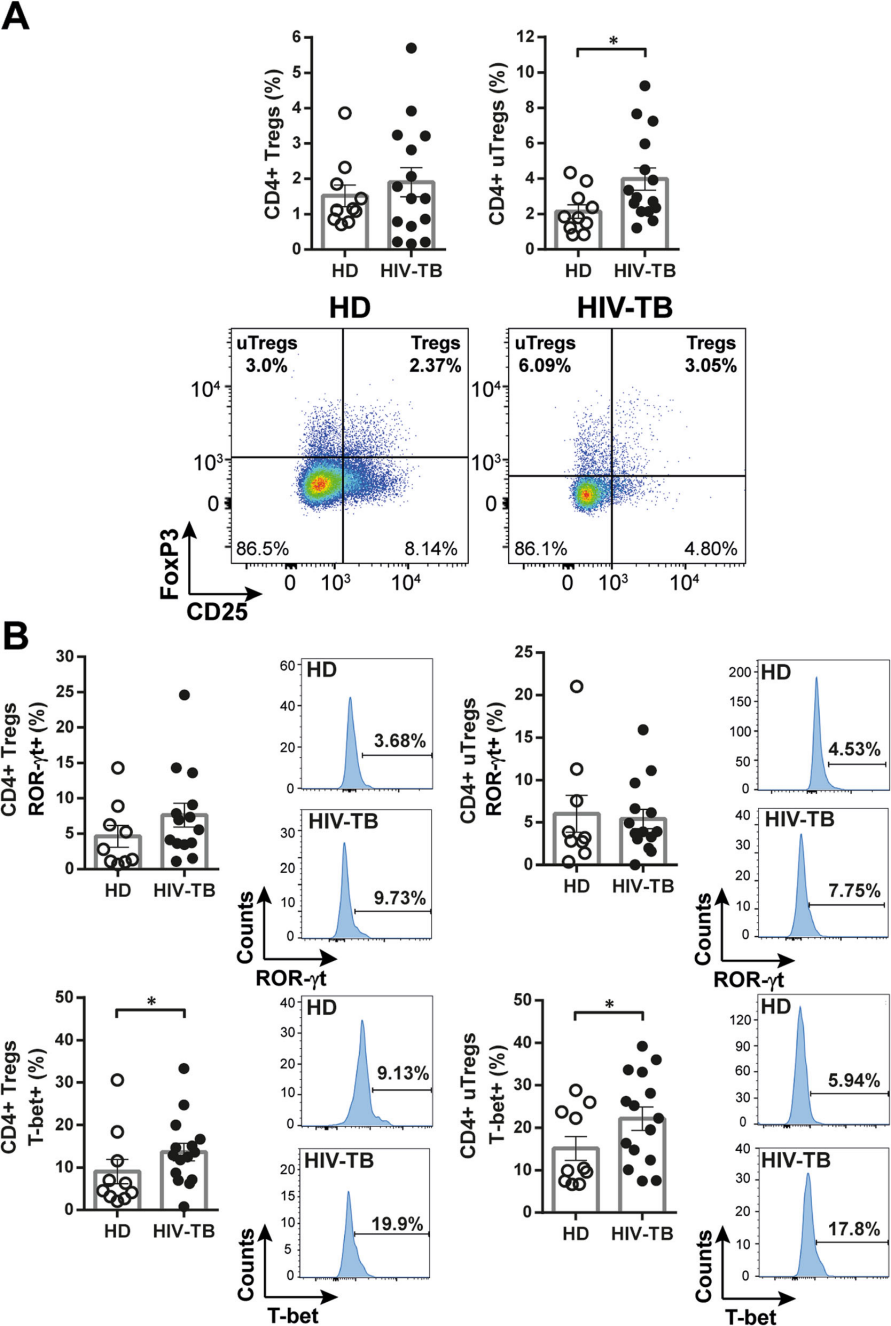
Analysis of Treg and uTreg subsets within CD4 + T cells from HIV-TB patients and HD. Recently thawed or freshly isolated PBMCs were stained and analyzed by flow cytometry. Figure shows the percentage of (**a**) CD4+ regulatory T cells (Tregs, CD25 + FoxP3+) and CD4+ unconventional regulatory T cells (uTregs, CD25-FoxP3+) and (**b**) the co-expression of the transcription factors ROR-γt or T-bet within each population. The results are plotted for HD (open circles) and HIV-TB patients (black circles). Each symbol represents an individual subject. Unpaired t test or Mann-Whitney U test, as appropriated *p < 0.05. Representative flow cytometry examples are shown

Determination of the expression of transcription factors (TF) FoxP3, ROR-γt and T-bet and of CD25 in PBMCs from HIV-TB patients and HD was performed. In ex-vivo analyses we found that HIV-TB patients presented higher frequencies of CD4+ T-bet+ and CD4 + FoxP3 + T-bet+ cells, and exhibited lower numbers of CD4+ ROR-γt + subpopulation compared to HD (Fig. 4a and b). Besides conventional regulatory CD4 + T cells (i.e. Tregs, CD25 + FoxP3+), the frequency of “unconventional” regulatory CD4 + T cells (i.e. uTregs, CD25-FoxP3+) was also analyzed. This is a CD4 + FoxP3+ T cell population with undetectable levels of CD25 [25] that exerts an inhibitory capacity supressing proliferation and cytokine production from effector T cells [26]. We found that in HIV-TB patients uTregs were expanded, as reported elsewhere (Fig. 5a and [25]). Moreover, within this cohort a larger frequency of T-bet+ cells were observed in both Tregs and uTregs populations (Fig. 5 b).

### The balance Th1/Tregs may play an essential role in clinical outcome

**Fig. 6:**
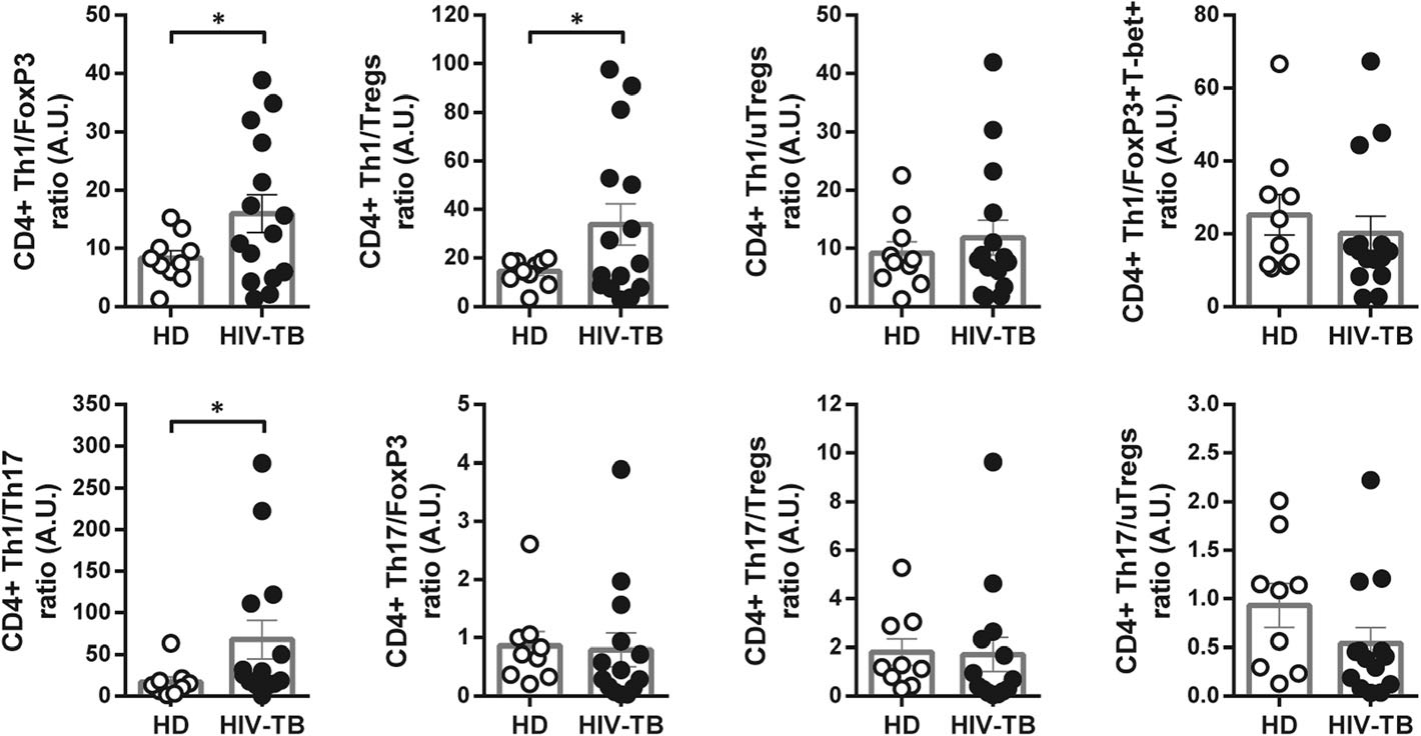
Changes in the balance between Th1, Tregs, uTregs and Th17 cells in patients with HIV-TB compared with HD. Recently thawed or freshly isolated PBMCs were stained and analyzed by flow cytometry. Figure shows CD4 + T cell subset ratios. The results are plotted for HD (open circles) and HIV-TB patients (black circles). Each symbol represents an individual subject. Unpaired t test or Mann-Whitney U, as appropriated *p < 0.05

**Fig. 7:**
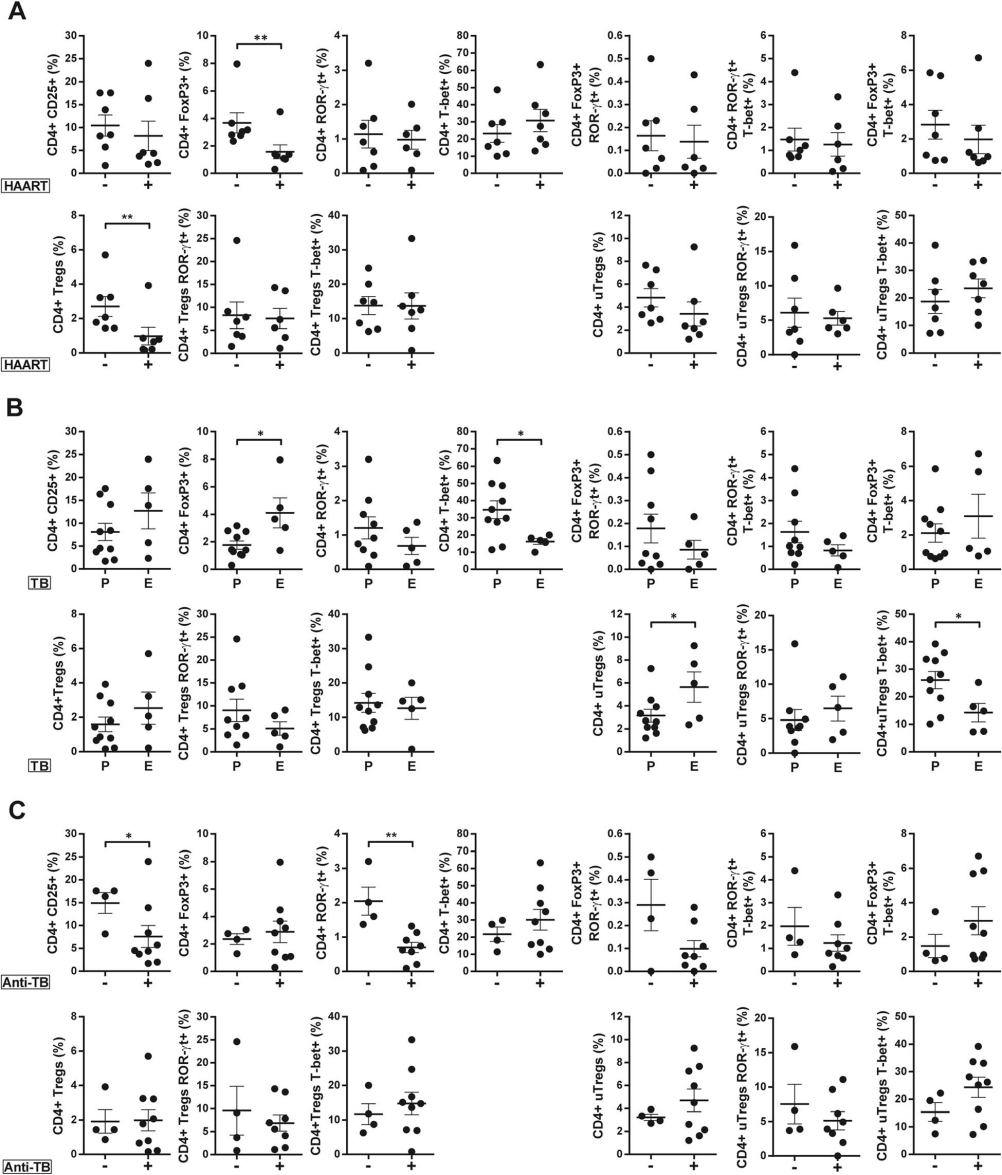
Analisys of different CD4 + T cell subsets and different clinical parameters from HIV-TB patients. CD4 + T cell phenotype from HIV-TB were associated with clinical data: (**a**) patients on HAART (+) vs. untreated patients (−) at the moment of sample collection, (**b**) diagnosis of pulmonary (P) or extrapulmonary (E) TB, and (**c**) individuals on anti-tuberculous treatment -less than two weeks- (+) vs. untreated patients (−) at the moment of sample collection. Each symbol represents an individual subject. Unpaired t test or Mann-Whitney U test, as appropriated *p < 0.05 and **p < 0.01

The balance among Th1, Th17, Tregs and uTregs phenotypes was analyzed. Unexpectedly, data revealed that HIV-TB individuals presented a higher proportion of Th1 cells over Tregs and Th17 populations, when compared to HD (Fig. 6). We contrasted our findings with clinical data from the patients enrolled. The frequency of CD25+, FoxP3+ or FoxP3 + T-bet+ cells was negatively associated with CD4 + T cell count. The same results were observed for uTregs and Tregs, which also correlated negatively with CD4/CD8 T cell ratio. CD4 + FoxP3+ cells were associated with lower nadir CD4 + T cell count and CD4/CD8 T cell ratio, whereas this subset presented a positive correlation with viral load (Table 2A). In addition, greater frequencies of CD4 + FoxP3+ T cells were found in patients who were neither on highly active antiretroviral therapy (HAART) nor TB treatment and in those with extrapulmonary TB. Similarly, higher proportions of Tregs were found in patients who were not receiving HAART, and uTregs were present to a greater extent in those individuals with disseminated TB (Fig. 7a and b). These results suggest that the expression of FoxP3 in CD4 + T cells may be associated with an unfavorable disease outcome in HIV-TB patients. Conversely, T-bet expression could be related to a favorable clinical outcome, since it was associated with greater CD4 + T cell counts and nadir value (Table 2A) and with the development of a lung-restricted TB instead of a disseminated infection. Moreover, uTregs which express T-bet were present in greater frequency in individuals with pulmonary TB (Fig. 7b). Finally, in individuals who had not received TB treatment, higher frequencies of CD4 + T cells expressing CD25 or ROR-γt were found (Fig. 7c).

**Table 2:** Correlation analysis between the transcriptional profile of *Mtb*-Specific CD4 + T cells and clinical data from HIV-TB patients. Table shows the results of CD4 + T cells expressing **(A)** CD25, FoxP3, ROR-γt or T-bet and the co-expression of FoxP3, ROR-γt and T-bet. Additionally, **(B)** Tregs (CD25 + FoxP3+) and uTregs (CD25-FoxP3+) subsets and the co-expression of the transcription factors ROR-γt or T-bet within each subpopulation were analyzed. A comparison of the percentage of different CD4+ Th cells was performed with total and nadir CD4 + T cell counts, CD4/CD8 ratio and viral load. Number of values (n), Spearman’s rank correlation coefficient (r), and *p* value are indicated

Clinical parameter	FoxP3+	ROR-*γ*t+	T-bet+	CD25+	FoxP3+ ROR-*γ*t+	ROR-*γ*t + T-bet+	FoxP3+ T-bet+
A							
CD4 + T cell count (cell/ml)	n = 14	*n* = 14	*n* = 15	*n* = 15	*n* = 14	*n* = 14	*n* = 15
	r = −0.66	r = 0.14	r = 0.48	r = − 0.45	r = 0.29	r = 0.37	r = − 0.49
	*p* = 0.0035	*p* = 0.31	*p* = 0.041	*p* = 0.045	*p* = 0.16	*p* = 0.19	*p* = 0.029
Nadir CD4 T cell count (cell/ml)	*n* = 15	*n* = 14	*n* = 14	*n* = 15	*n* = 14	*n* = 14	*n* = 15
	r = −0.65	r = 0.55	r = 0.65	r = − 0.41	r = 0.091	r = 0.61	r = − 0.29
	*p* = 0.021	*p* = 0.45	*p* = 0.014	*p* = 0.22	*p* = 0.40	*p* = 0.033	*p* = 0.28
CD4/CD8 T cell ratio	*n* = 15	*n* = 14	*n* = 15	*n* = 15	*n* = 14	*n* = 14	*n* = 15
	r = −0.55	r = − 0.12	r = 0.23	r = − 0.27	r = − 0.034	r = 0.13	r = − 0.17
	*p* = 0.018	*p* = 0.35	*p* = 0.21	p = 0.16	*p* = 0.49	*p* = 0.63	*p* = 0.27
Viral load (copies/ml)	*n* = 15	*n* = 14	*n* = 15	*n* = 15	*n* = 14	*n* = 14	*n* = 15
	r = 0.56	r = 0.25	r = − 0.14	r = 0.41	r = 0.15	r = 0.013	r = 0.071
	*p* = 0.015	p = 0.19	*p* = 0.32	*p* = 0.13	*p* = 0.30	*p* = 0.97	*p* = 0.80
B							
Clinical parameter	Tregs	Tregs ROR-*γ*t+	Tregs T-bet+	uTregs	uTregs ROR-*γ*t+	uTregs T-bet+	
CD4 + T cell count (cell/ml)	*n* = 15	*n* = 14	*n* = 15	*n* = 15	*n* = 14	*n* = 15	
	r = −0.71	r = 0.33	r = − 0.17	r = − 0.48	r = 0.059	r = − 0.22	
	*p* = 0.015	*p* = 0.25	*p* = 0.54	*p* = 0.036	p = 0.84	*p* = 0.42	
Nadir CD4 T cell count (cell/ml)	*n* = 15	*n* = 15	*n* = 14	*n* = 14	*n* = 14	*n* = 14	
	r = − 0.43	r = 0.16	r = − 0.19	r = − 0.29	r = − 0.27	r = − 0.22	
	*p* = 0.090	*p* = 0.66	*p* = 0.58	*p* = 0.19	*p* = 0.44	p = 0.49	
CD4/CD8 T cell ratio	*n* = 15	*n* = 14	*n* = 15	*n* = 15	*n* = 14	*n* = 15	
	r = − 0.53	r = 0	r = − 0.17	r = − 0.27	r = − 0.059	r = − 0.16	
	*p* = 0.022	*p* = 0.50	*p* = 0.55	*p* = 0.15	*p* = 0.84	*p* = 0.55	
Viral Load (copies/ml)	*n* = 14	*n* = 14	*n* = 14	*n* = 14	*n* = 14	*n* = 14	
	r = 0.42	r = 0.0083	r = − 0.23	r = 0.17	r = − 0.033	r = − 0.14	
	*p* = 0.14	r = 0.98	*p* = 0.43	p = 0.27	*p* = 0.92	p = 0.63

**Fig. 8:**
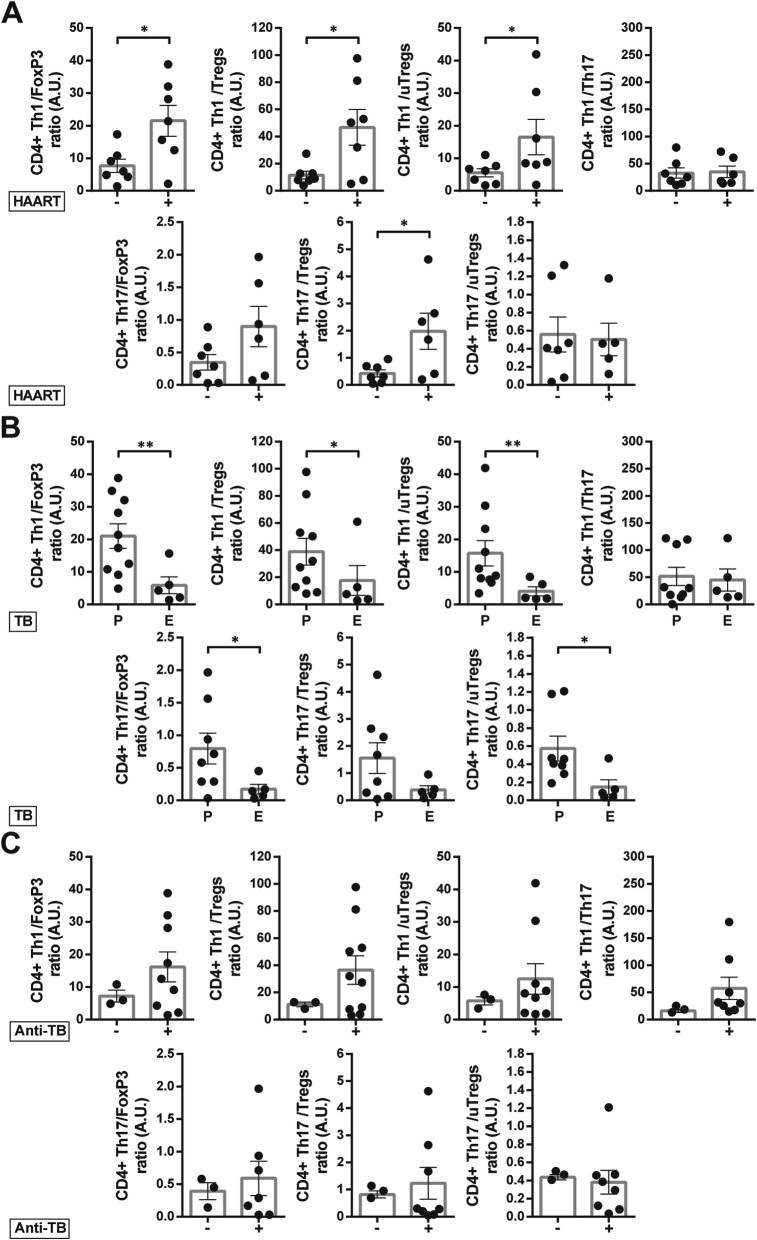
Association between the CD4 + T cell subsets ratio with clinical parameters in HIV-TB patients. Phenotype of CD4 + T cell subset ratios from HIV-TB were correlated with clinical data: (**a**) patients on HAART (+) vs. untreated patients (−) at the moment of sample collection, (**b**) diagnosis of pulmonary (P) or extrapulmonary (E) TB, and (**c**) individuals on anti-tuberculous treatment -less than two weeks- (+) vs. untreated patients (−) at the moment of sample collection. Each symbol represents an individual subject. Unpaired t test or Mann-Whitney U test, as appropriated *p < 0.05 and **p < 0.01

The association between clinical data and the ratio among Th1, Th17, Tregs, uTregs and CD4 + Foxp3+ T subsets was also analyzed. A higher Th1/FoxP3 ratio was related with greater CD4 + T cell counts and nadir values. Furthermore, Th1/FoxP3, Th1/Tregs and Th1/uTregs ratios were increased in patients on HAART and diagnosed with lung-restricted TB infection (Fig. 8a and b). In addition, a greater Th1/Tregs balance positively correlated with total and nadir CD4 + T cell counts, CD4/CD8 T cell ratio and had negative association with viral load (Table 3). On the other hand, Th17/FoxP3 ratio positively correlated with greater values of total and nadir CD4 + T cell counts and with pulmonary TB (Table 3). Moreover, Th17/Treg ratio was associated with higher CD4 + T cell counts and individuals on HAART. Lastly, Th17/uTregs ratio was greater in HIV-TB patients with pulmonary TB compared with those with a disseminated disease. No associations were found between the balance of CD4+ Th subpopulations and anti-TB treatment, probably due to the low number of untreated patients at the time of recruitment (Fig. 8b).

**Table 3:** Correlation between clinical parameters and ratios of different CD4+ subpopulations. A comparison of different CD4+ subsets ratios was performed with total and nadir CD4 + T cell counts, CD4/CD8 ratio and viral load. Number of values (n), Spearman’s rank correlation coefficient (r), and p value are indicated

Clinical parameter	Th1/ FoxP3	Th1/ Tregs	Th1/ uTregs	Th1/ Th17	Th17/ FoxP3	Th17/ Tregs	Th17/ uTregs
CD4 + T cell count (cell/ml)	*n* = 15	*n* = 14	*n* = 15	*n* = 14	*n* = 13	*n* = 13	*n* = 13
	r = 0.59	r = 0.70	r = 0.55	r = 0.055	r = 0.57	r = 0.65	r = 0.24
	*p* = 0.011	*p* = 0.0026	*p* = 0.018	*p* = 0.43	*p* = 0.024	*p* = 0.18	*p* = 0.44
Nadir CD4 T cell count (cell/ml)	*n* = 15	*n* = 14	*n* = 15	*n* = 14	*n* = 14	*n* = 14	*n* = 14
	r = 0.60	r = 0.66	r = 0.36	r = 0.37	r = 0.56	r = 0.48	r = 0.20
	*p* = 0.025	*p* = 0.018	*p* = 0.14	*p* = 0.15	*p* = 0.048	*p* = 0.16	*p* = 0.58
CD4/CD8 T cell ratio	*n* = 15	*n* = 14	*n* = 15	*n* = 14	*n* = 13	*n* = 13	*n* = 13
	r = 0.25	r = 0.49	r = 0.20	r = 0.23	r = 0.22	r = 0.33	r = 0.14
	p = 0.19	*p* = 0.036	*p* = 0.23	*p* = 0.21	*p* = 0.23	*p* = 0.26	*p* = 0.65
Viral Load (copies/ml)	*n* = 15	*n* = 15	*n* = 15	*n* = 14	*n* = 14	*n* = 14	*n* = 14
	r = −0.37	r = −0.51	r = − 0.38	r = − 0.34	r = − 0.25	r = − 0.28	r = 0.19
	*p* = 0.087	*p* = 0.027	*p* = 0.081	*p* = 0.12	*p* = 0.19	*p* = 0.33	*p* = 0.52

### *Mtb* alters CD4+ T cell phenotype in vitro

**Fig. 9:**
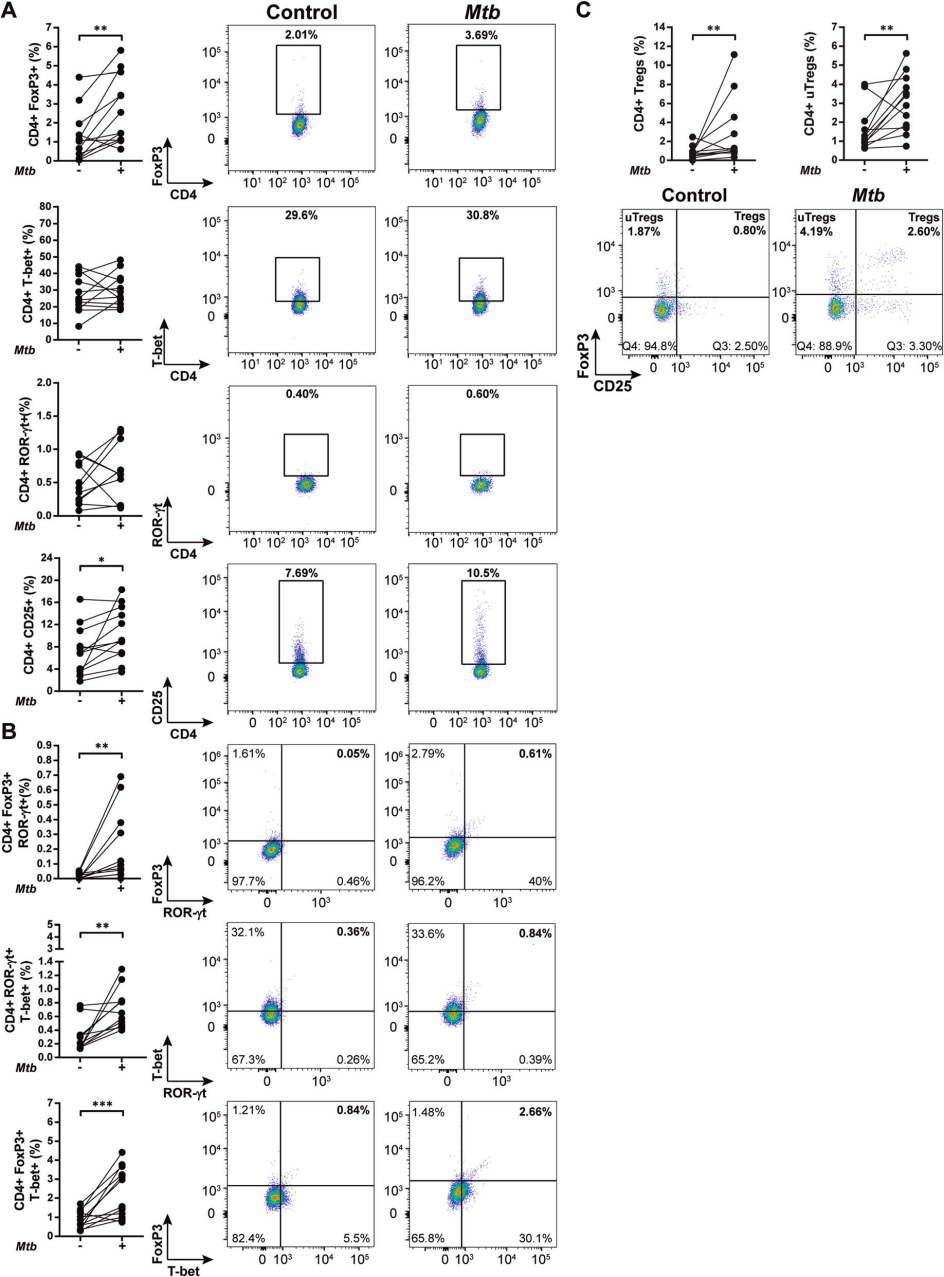
*Mtb* induces changes in CD4+ T-cell phenotype from HIV-TB patients. Recently thawed or freshly isolated PBMCs from HIV-TB individuals were stimulated with an antigen of *Mtb,* stained and analyzed by flow cytometry, as indicated in methods. Figure shows the percentage of CD4 + T cells that express (**a**) FoxP3, T-bet, ROR-γt or CD25 and (**b**) the co-expression of transcription factors using a Boolean gating strategy. **c** Tregs (CD4+ CD25+ FoxP3+) and uTregs (CD4+ CD25- FoxP3+) subsets were also analyzed. Representative flow cytometry examples are shown. Each symbol represents an individual subject. Unpaired t test or Mann-Whitney U test, as appropriated *p < 0.05, **p < 0.01 and ***p < 0.005

The capacity of *Mtb* to modulate the profile of CD4+ T cell subpopulations in vitro was analyzed. *Mtb* stimulation increased the frequency of CD4 + CD25+ and CD4 + FoxP3 cells, but it did not modify the proportion of ROR-γt and T-bet in HIV-TB patients (Fig. 9 a). The same pattern was detected in HD, with the exception of greater proportion of CD4 + T-bet+ (Additional file 2: Figure S2 A). Besides, *Mtb* increased the frequency of CD4 + T cells that expressed two transcription factors simultaneously in both HIV-TB and HD (Fig. 9 b and Additional file 2: Figure S2 B). In addition, *Mtb*-stimulation induced a higher expression of CD25 on a per cell basis and downregulated ROR-γt expression (Additional file 5: Table S1) in both study cohorts.

**Fig. 10:**
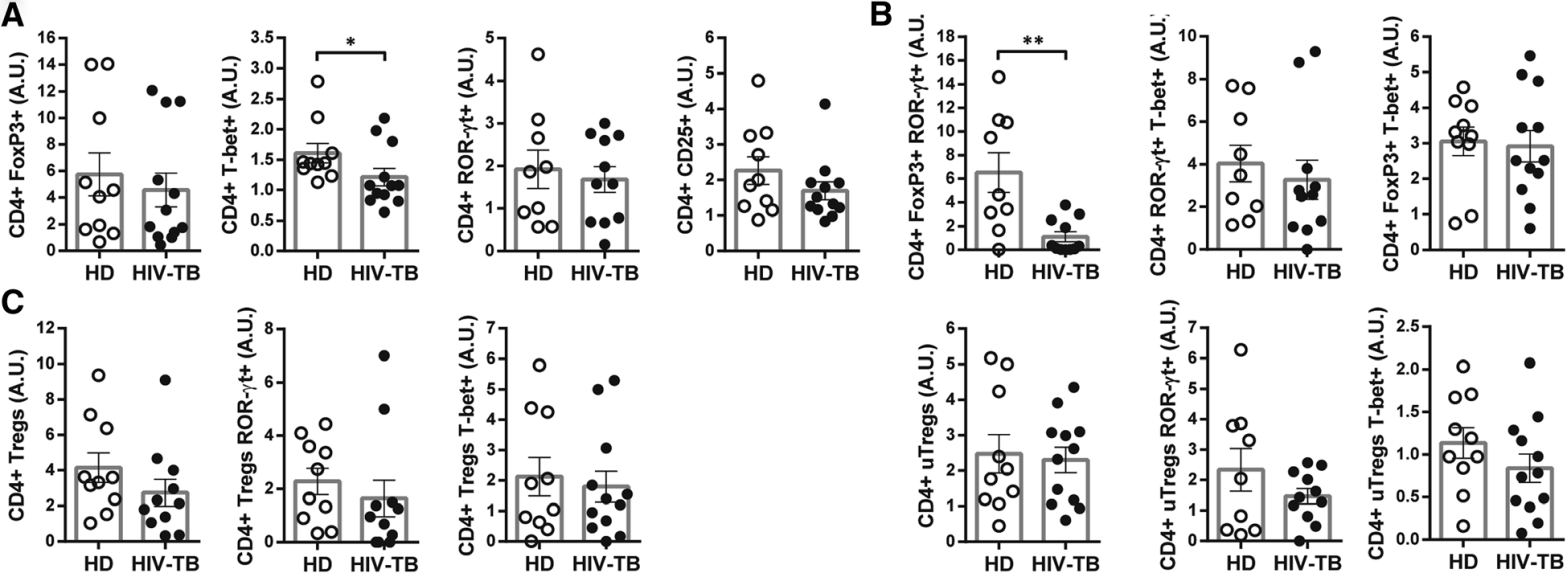
Comparative CD4 + T cell response from HD and HIV-TB cohorts to *Mtb*. Recently thawed or freshly isolated PBMCs from HD and HIV-TB individuals were stimulated with an antigen of *Mtb,* stained and analyzed by flow cytometry, as indicated in methods. The results are plotted for HIV-TB (black circles) patients and HD (open circles). Each symbol represents an individual subject. The figure shows the percentage of CD4 + T cells that express (**a**) FoxP3, T-bet, ROR-γt or CD25 and (**b**) the co-expression of transcription factors using a Boolean gating strategy. (**c**) Tregs (CD4 + CD25 + FoxP3+) and uTregs (CD4 + CD25-FoxP3+) subsets and the co-expression of ROR-γt or T-bet within each subpopulation were analyzed. Values are relativized to unstimulated cells. Error bars show mean ± SEM. Unpaired t test or Mann-Whitney U test, as appropriated *p < 0.05 and **p < 0.01

*Mtb* stimulation also augmented the frequency of Tregs and uTregs in both HIV-TB individuals and HD (Fig. 9c and Additional file 2: Figure S2 C). However, there were no changes in the proportion of cells that expressed ROR-γt and T-bet within these populations (data not shown). Finally, we contrasted the changes induced by *Mtb* between groups and observed significant differences in the frequency of CD4+ T-bet+ and CD4 + FoxP3 + ROR-γt + cells, finding a greater proportion of these populations in HD (Fig. 10).

### 7-OD modifies *Mtb*-specific immune response in the context of HIV and *Mtb* infection

**Fig. 11:**
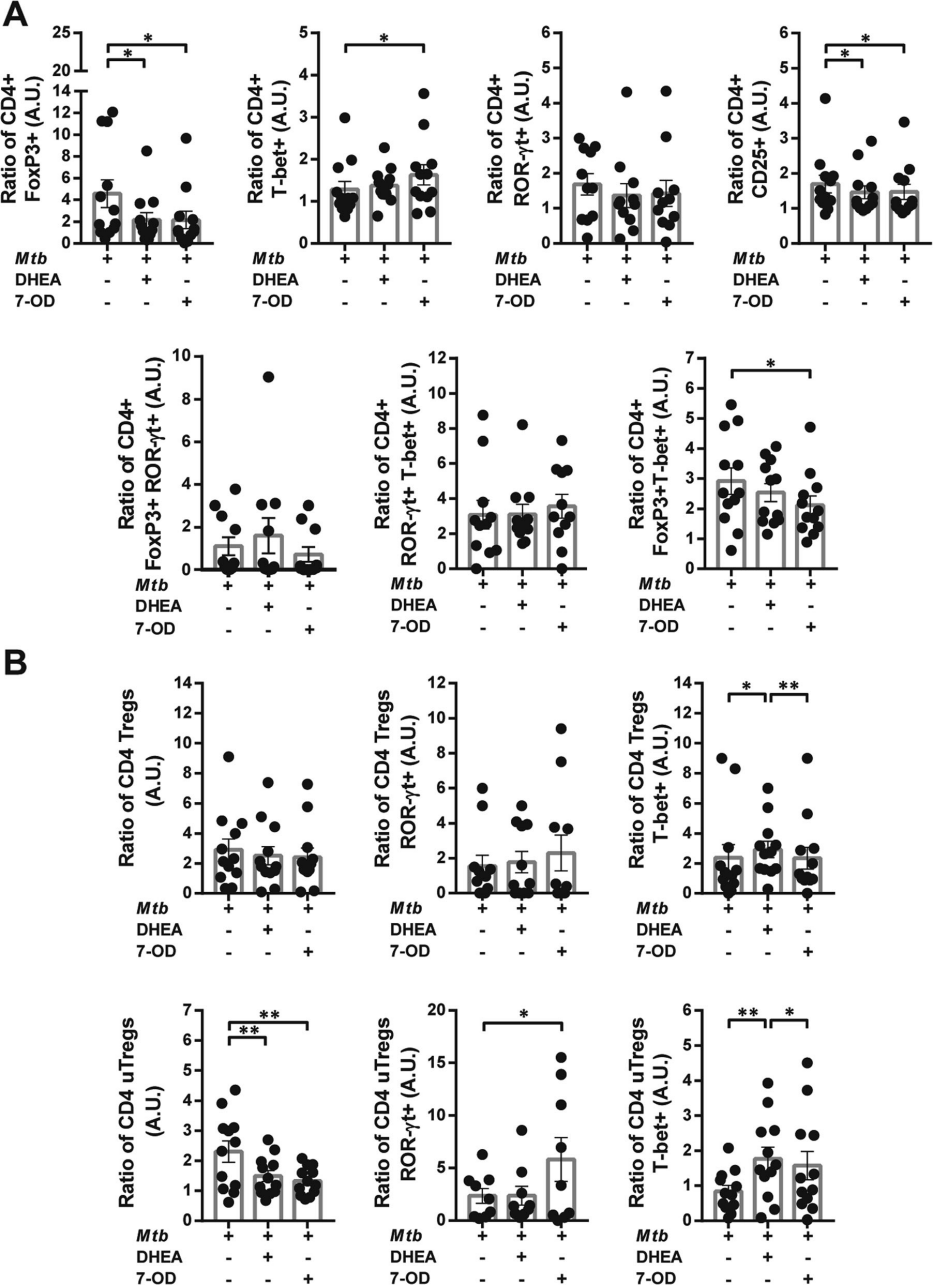
*Mtb*-specific response in HIV-TB patients is modulated differentially by 7-OD and DHEA. Recently thawed or freshly isolated PBMCs from HIV-TB patients were stimulated with an antigen of *Mtb* in the presence/absence of 7-OD at 1 × 10^−6^M or DHEA at 1 × 10^−7^M. Then, cells were stained and analyzed by flow cytometry, as described before. This figure shows the percentage of CD4 + T cells that express (**a**) FoxP3, T-bet, ROR-γt or CD25 and the co-expression of transcription factors using a Boolean gating strategy or (**b**) CD4+ Tregs and uTregs, with the co-expression of the transcription factors ROR-γt or T-bet within each population. Values are relativized to unstimulated cells. The results are plotted for HIV-TB patients. Each symbol represents an individual subject. Friedman test followed by post-hoc comparisons: Fisher’s LSD or Dunn’s test, as appropriated *p < 0.05, **p < 0.01 and ***p < 0.005

As 7-OD modulated *Mtb*-specific lymphoproliferation and cytokine production, we aimed to study its effect on CD4+ Th cell phenotype. Remarkably, 7-OD increased the frequency of CD4+ T-bet and reduced the frequency of CD4 + FoxP3 + T-bet+ in HIV-TB patients. Furthermore, both 7-OD and DHEA diminished the percentage of cells expressing CD25+ or FoxP3+, and restored the effect of *Mtb* on CD4+ lymphocytes (Fig. 11a). Within the cohort of HD, while 7OD raised the frequency of CD4+ T-bet+ cells, DHEA expanded the CD4 + FoxP3 + RORγ-t + population and declined the number of cells that co-expressed FoxP3+ and T-bet after antigen (Ag)-exposure (Additional file 3: Figure 3A).

Concerning regulatory populations, treatment with both 7-OD and DHEA decreased the frequency of uTregs without modifying Tregs in HIV-TB individuals (Fig. 11b), while both Tregs and uTregs in HD declined in the presence of DHEA (Additional file 3 Fig. S3B). Besides, DHEA enhanced the expression of T-bet in Tregs and uTregs in the group of HIV-TB (Fig. 11b), while 7-OD augmented the proportion of uTregs expressing ROR-γt in HIV-TB and HD (Fig. 11b and Additional file 3: Figure S3B). Together, these data suggest that both 7-OD and DHEA are able to modify the effect of *Mtb* on CD4+ Th cell phenotype, but only 7-OD modulates it toward a Th1-like profile.

The expression of each TF was also analyzed on a per cell basis. Our data reveals that 7-OD enhanced the levels of T-bet in CD4 + T cells from HIV-TB (Additional file 5: Table S1). However, this did not occur in HD individuals (data not shown). In conclusion, 7-OD not only modified the percentage of cells expressing different TF, but also changed their levels of expression in HIV-TB patients, partially restoring the effect of *Mtb* on these populations.

### The balance among different CD4 + T cell subpopulations is disturbed by *Mtb* and modified by 7-OD and DHEA

**Fig. 12:**
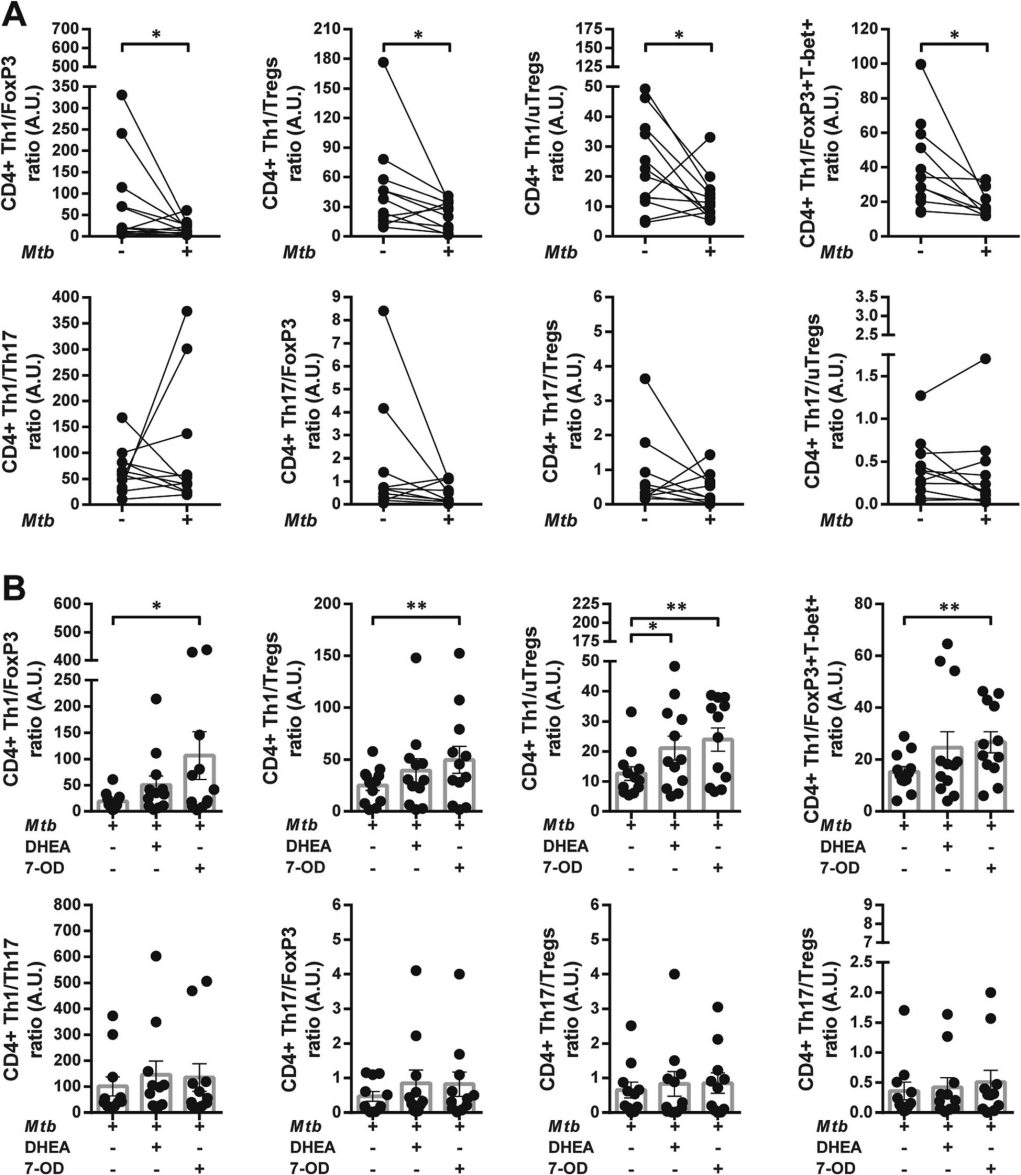
The equilibrium between Th1, Tregs, uTregs and Th17 subsets are disrupted by *Mtb* and modified by 7-OD and DHEA. Recently thawed or freshly isolated PBMCs from HIV-TB individuals were stained and analyzed by flow cytometry, as indicated in methods. Figure shows CD4 + T cell subset ratios. The results are plotted for HIV-TB patients (black circles) comparing (**a**) Control vs. stimulated cells. Unpaired t test or Mann-Whitney U test, as appropriated *p < 0.05. (**b**) Distinct CD4+ Th subset ratios of *Mtb*-stimulated PBMCs treated with 7-OD and DHEA. Each symbol represents an individual subject. Friedman test followed by post-hoc comparisons: Fisher’s LSD or Dunn’s test, as appropriated *p < 0.05, **p < 0.01 and ***p < 0.005

In order to study the changes that *Mtb* induced on the CD4 + T cell phenotype, TF ratio among different subpopulations was analyzed. The stimulation of PBMCs with *Mtb* reduced Th1/FoxP3, Th1/Tregs and Th1/FoxP3 + T-bet+ ratios in both HD and HIV-TB cohorts (Fig. 12a and Additional file 4: Figure S4A). Additionally, in co-infected individuals, Th1/uTregs ratio was also decreased (Fig. 12a). No variations in Th1/Th17 and Th17/regulatory ratios were observed in coinfected patients (Fig. 12a), but we found that *Mtb* reduced Th17/FoxP3 balance in HD (Additional file 4: Fig. S4A).

We wondered if the unbalance in CD4+ Th cells elicited by *Mtb* may be restored by in vitro treatment with 7-OD and/or DHEA. Remarkably, 7-OD induced an increase in Th1 population over CD4+ FoxP3+, Tregs, uTregs and FoxP3 + T-bet+ T cells in HIV-TB patients. Additionally, DHEA also raised Th1/uTregs ratio in co-infected individuals (Fig. 12b). On the other hand, within the group of HD, 7-OD augmented Th1/FoxP3 and Th1/Tregs ratios while DHEA had the same effect on Th1/uTregs balance. Lastly, in the same cohort, DHEA diminished Th1/Th17 ratio (Additional file 4: Figure S4B). These data demonstrate that CD4 + T cells develop a specific immunity toward certain Th phenotype through interactions with *Mtb* antigens, associated with a regulatory pathway. Nevertheless, this immunosuppressive milieu was overturn by the influence of 7-OD, which allowed the appropriate expansion of Th1 CD4 + T cells, an important subset for disease resolution.

## Discussion

HIV deleterious effects over TB infection are diverse, involving the specific and lasting effect on *Mtb*-specific immunity. The type of individual immune response will determine disease progression or its development into a chronic stage. Novel host-directed therapies (HDT) that potentiate immunity could be discovered by studying and identifying factors that lead to disease progression or resolution. Therefore, the aim of this research was to scrutinize the role of 7-OD on lymphocyte phenotype and function during HIV-TB coinfection.

The ability to proliferate and differentiate into cytokine-producing effector cells is an important characteristic to pathogen control [27]. We found that patients exhibited higher proliferative response compared to HD, which can be explained by *Mtb-*specific T cell clones in HIV-TB. HD responded to Ag-stimulation owing to previous BCG immunization or exposure to environmental mycobacteria [28]. Previous reports also demonstrated HIV-TB subjects maintain *Mtb*-specific proliferative capacity, regardless of the low absolute CD4 + T cells counts and the rapid depletion of *Mtb*-specific responses after HIV infection [23, 29]. The current results show that 7-OD induces an augmented lymphoproliferation, while DHEA seems to suppress this activity. The lack of modulation by DHEA was reported formerly in PBMCs from TB patients [30]. Thus, our data indicate that concentrations of 7-OD superior to a physiologic range may enhance *Mtb*-specific immune lymphoproliferative response during HIV-TB coinfection.

Because of inter-individual variability, the use of cytokine ratios rather than single cytokine measurements would improve the study of immune responses. We demonstrate that the production of the protective cytokines IFN-γ and TNF-α is accompanied by the secretion of IL-17A in confected individuals, suggesting a heightened cytokine inflammatory response. Interestingly, HIV-TB individuals displayed reduced IFN-γ/IL-10, IFN-γ/IL-17A and IFN-γ/TNF-α ratios compared to HD. Other authors described a lower ratio of IFN-γ/IL-10 in drug-resistant patients during TB-Immune Reconstitution Inflammatory Syndrome (IRIS), which correlated with higher bacterial load [31]. In systemic lupus erythematosus, it was reported that TNF-α could be protective, since its levels and TNF-α/IL-10 ratio were lower in patients with active disease [32]. Furthermore, it was found higher IFN-γ/TNF-α ratio in household contacts when compared to TB patients [33]. Remarkably, 7-OD modified the impaired immune response against *Mtb* in HIV-TB patients, with an augment in IFN-γ and TNF-α secretion as well as an increment in IFN-γ/IL-10 and TNF-α/IL-10 ratios. The effect of 7-OD in lymphoproliferation and cytokine production in response to *Mtb* may induce a protective Th1-like response in the context of HIV-TB coinfection.

Then, we investigated CD4 + T cell phenotype of PBMCs from HD and patients ex vivo. We observed that HIV-TB individuals exhibited lower frequencies of CD4+ ROR-γt + T cells but greater proportions of CD4 + T-bet+, CD4 + FoxP3 + T-bet+ and uTregs (CD4 + CD25-FoxP3+). We found that higher proportions of the CD4 + ROR-γt + subset were associated with those patients on TB treatment vs. untreated individuals. Although its role in human pathology has not yet been fully elucidated, a mouse model revealed that cells expressing ROR-γt + were critical to the induction of an *Mtb*-specific memory response [34, 35]. Additionally, we observed larger frequencies of the CD4 + T-bet+ population that were associated with better clinical status and propitious disease outcome. These results are in line with the role of T-bet as the master regulator of Th1 cells, involved in the protective and inflammatory responses during *Mtb* infection [7, 24]. Conversely, we associated both uTregs and CD4 + FoxP3 + T-bet+ T cells with an unfavorable outcome. Furthermore, uTregs were found in greater proportion in those individuals with disseminated TB. A recent study revealed that a cohort of HIV+ individuals with latent TB exhibited a reduction in CD4 + FoxP3 + T-bet+ subset, which correlated inversely with viral load [27]. Our results indicated that this population is associated with an unfavorable result, but differences may be due to a different recruited cohort and that the authors studied Ki67+ cells CD4 + T cells [36]. Otherwise, our previous results observed an increased frequency of uTregs during HIV-TB disease [25] and described that this population exerts regulatory functions, in spite of an altered surface expression of markers and differences in cytokine production [37]. In line with this, lower Th1/uTregs ratio was observed in those patients who were not on HAART and who developed a disseminated TB infection. Our data therefore suggest that the expansion of uTregs population may be involved in the pathogenesis of TB.

Ex vivo analysis also showed that Th1/FoxP3 and Th1/Tregs ratios were higher in HIV-TB cohort. However, superior proportion of Th1 cells as well as Th1/FoxP3 and Th1/Tregs ratios positively correlated with total and nadir CD4 + T cell counts and with those individuals who presented pulmonary instead of disseminated TB. Similarly, higher proportions of Th1 population over CD4 + FoxP3+ and Tregs were associated with patients on HAART, while Th1/Tregs were related with a higher CD4/CD8 T cell ratio and negatively correlated with viral load. We hypothesize that the balance between Th1 and FoxP3+ or Tregs cells may have a critical role in the immune response during HIV-TB, as these parameters are related with clinical data associated with disease consequences, as we discussed in a previous report [17].

Afterwards, we analyzed in vitro *Mtb* stimulation on CD4+ Th phenotype. Ag-stimulation raised the proportion of CD4 + FoxP3+, Tregs and uTregs in HIV-TB patients. The proportion of Treg in pleural fluid inversely correlated with *Mtb*-specific immunity at the site of infection [38]. Higher frequencies of Treg suggest that these cells participate in immunosuppression observed in individuals with more severe active disease [38, 39] and persist beyond completion of anti-TB therapy [40]. Furthermore, in HIV-TB patients we observed a significant drop in frequencies of uTregs in response to TB treatment (unpublished data). While a Th1 response orchestrated immunity to intracellular pathogens, CD4 + T cells with a regulatory phenotype were associated with long-term adverse outcomes [27, 41]. Therefore, the balance between effector and suppressive immune responses is essential to control *Mtb* infection.

Hormone effect on CD4 + T cell lineage in a HIV-TB milieu was studied. Of note, 7-OD reduced the frequency of CD4 + T cells with a regulatory function and led to an augment in the frequency of CD4 + T-bet+ subset. This hormone also increased the proportion of cells expressing ROR-γt within uTregs. Although we could not find any association between the former population and clinical data, studies performed in mice and humans demonstrated that Tregs can be reprogrammed into IL-17 + Foxp3+ T cells, which are believed to control pathological inflammation, potentially antagonizing pro-inflammatory IL-17-producing cells [42].

There may be an association between a transcription factor or marker expression levels and a functional capacity [43, 44]. The assessment of MFI revealed that *Mtb*-stimulation induced higher levels of CD25 and downregulated ROR-γt expression on total CD4 + T cells. A research work found that ROR-γt expression was diminished in tuberculin skin test (TST) positive individuals, suggesting that lack of Th17 cells predisposes to latent infection [45]. At the same time, an augment in the expression of CD25 was associated with a regulatory phenotype in the context of TB [40, 46] and HIV [47]. Our data demonstrated that 7-OD modified the effect of *Mtb on* CD4+ lymphocytes by augmenting the expression of ROR-γt and T-bet, potentially enhancing their immune functions. In summary, *Mtb* boosts the frequency of CD4 + T cells that expresses FoxP3 and modified the expression of CD25 and ROR-γt on a per cell basis. However, 7-OD could reverse this effect by changing the frequency of effector Th1 cells and regulatory CD4 + T cells and the levels of transcription factors, improving thus its functionality.

It is known that *Mtb* infection induces highly polyfunctional responses [48], stimulating the co-expression of FoxP3, T-bet and/or ROR-γt in CD4 + T lymphocytes and that HIV infection alters the transcriptional profiles of these cells [27]. Our findings showed that exposure to *Mtb* antigens promoted the expansion of those populations in HD and coinfected patients. Cells producing both IL-17 and IL-10 were considered to play a protective regulatory homeostatic role, while CD4+ Th17 population co-secreting IFN-γ were associated with pathology [49]. CD4 + FoxP3 + ROR-γt + lymphocytes have the capacity to produce IL-17 and exhibit suppressive functions via a cell-cell contact mechanism and the secretion of IL-10 [42, 50]. In subjects with latent TB significantly higher frequencies of regulatory IL10 + Th17 were found compared to subjects with extrapulmonary TB when PBMCs were stimulated with latency antigens. Conversely, the response to ESAT6/CFP10 was predominantly managed by IFN-γ + Th17 T cells [49]. In this report, we found that higher frequencies of ROR-γt + T-bet+ CD4 + T cells in the cohort of HIV-TB patients were associated with greater levels of nadir CD4 + T cell counts, although we could not find any effects after the addition of hormones.

Recent studies demonstrated that CD4 + T cells expressing both, T-bet and FoxP3, exert regulatory rather than pro-inflammatory functions. T-bet is required for the expansion of these immunosuppressive cells to limit Th1-mediated immune responses [51, 52]. We hypothesized this subset might be involved in pathogen-specific loss of host resistance. In this report we found that *Mtb* augmented the proportion of CD4 + FoxP3 + T-bet+ cells, which was negatively associated with peripheral CD4 + T cell numbers, whereas treatment with 7-OD reduced the frequency of this subpopulation. Thus, the results indicate that 7-OD promotes an immunoprotective profile, not only by increasing the number of Th1 cells and the expression of T-bet per cell, but also by decreasing specific Th1 inhibitory (i.e., CD4 + FoxP3 + T-bet+ cells) responses.

Finally, we examined Th profile ratios inasmuch as the balance between Th1 and regulatory pathways might define resolution of *Mtb* infection and disease outcome. The role of Th cells has been investigated from active disease to post-treatment status [41, 53]. One report showed a reduction in Th1 cells in active-TB patients, followed by an increase in these cells after clinical cure [54]. Another report informed about an increase in the percentage of *Mtb*-specific Tregs during active TB, with lesser Th1/Treg and Th17/Treg ratios [54]. We observed that *Mtb* stimulation increased the numbers of lymphocytes with a regulatory phenotype over Th1 CD4 + T cells (lower Th1/FoxP3, Th1/Tregs, Th1/uTregs and Th1/FoxP3 + T-bet+ ratios in patients). Other authors suggested that it would be useful for monitoring progression of infection to focus on the study of the relative balance between Th1/regulatory cells rather than on the analysis of a specific cell subset [55]. We discovered 7-OD and DHEA augmented Th1/uTregs ratio, but only 7-OD could reverse the effect of *Mtb* in terms of the balance of Th1/FoxP3, Th1/Tregs and Th1/FoxP3 + T-bet+ in HIV-TB individuals.

There is little prior information about the mechanism of action of 7-OD on eukaryotic cells, but no data about its effects on CD4+ T lymphocytes. It was reported that 7-OD modulated cell metabolic activity by rising the levels of mitochondrial glycerol-3-phosphate dehydrogenase (GPD2) and malic enzyme in rats [56, 57]. Also, it was demonstrated that GPD2 participates in glycolysis, gluconeogenesis, glycerol and triacylglycerol metabolism [58]. Moreover, GPD2 is an alternative source of mitochondrial reactive oxygen species (mROS) [59], which are required for CD4+ T cell activation and expansion [60]. It was shown that reduction of GPD2 levels leads to decrease in ROS-mediated oxidative signal and the induction of NF-κB-dependent gene expression [59]. Likewise, NF-κB activation in T cells promoted clonal expansion during the Th1 response, Th1 differentiation and production of IL-2, IFN-γ and TNF-α [61, 62]. We therefore hypothesize that 7-OD stimulates GPD2 abundance and/or activity conducing to ROS production, NF-κB genes expression and the differentiation of CD4+ T cells to a Th1 subset. However, further research is needed to corroborate this hypothesis, given the unexplored nature of the effects of 7-OD on this cell type. Taking into account our results and the information from other authors, we can infer that both mechanisms are working in patients, since the effect at the cellular as well as the systemic level are operating. Thus, we hypothesize that 7-OD-induced modulation of 11β-HSD1 activity [63] from peripheral tissues could regulate cortisol levels and, in parallel, 7-OD stimulation of GPD2 on CD4+ T cells could impact on its metabolism, enhancing T cell activation, proliferation and Th1 differentiation. The differential effect of 7-OD in patients and HD may be caused by a higher number of *Mtb*-specific clones in HIV-TB individuals and/or a different metabolic state in CD4+ T cells that make them suitable to be modulated by 7-OD.

## Conclusions

In summary, our results suggest that *Mtb* modulate CD4 + T cell phenotype and functionality in HIV-TB patients. Besides, we described the effect of 7-OD on lymphoproliferation, cytokine production profile and CD4+ Th cell subpopulations in vitro. Firstly, 7-OD augmented the *Mtb*-induced proliferative capacity of PBMCs from the study population. Secondly, this hormone enhanced IFN-γ and TNF-α production, without suppressing the necessary action of IL-10 and IL-17A. Moreover, 7-OD promoted the secretion of IFN-γ and TNF-α over IL-10, therefore modifying the cytokine balance towards a more favorable profile for *Mtb* control. Third, while *Mtb* promoted a regulatory CD4 + T cell phenotype, 7-OD expanded CD4 + T-bet+ subpopulation and enhanced T-bet expression, which were related to improved clinical outcomes. Furthermore, 7-OD decreased the percentage of CD4 + T cells with immunosuppressive functions. Finally, 7-OD increased Th1/regulatory ratio, indicating the predominance of a Th1 response which may contribute to TB protection by secreting IFN-γ and activating anti-mycobacterial mechanisms. These results provide new data to delineate novel strategies that accompany the treatment of TB, by modulating T cell-mediated immunity against *Mycobacterium tuberculosis*.

## Supplementary information

**Additional file 1: Figure S1.** A representative analysis of the gating strategy developed during this study. Recently thawed or freshly isolated PBMCs were stained and analyzed by flow cytometry. The results are plotted for a sample from a HD labelled with (A) fluorochrome-conjugated Abs or (B) isotype control mAbs. Lymphocyte subset data were generated using a FSC-A/SSC-A gate. After doublet exclusion (FSC-A/FSC-H and FSC-H/FSC-W), live cells were selected using Live/Dead viability probe. Then, CD3 + CD4+ cells were gated and within this group, a gating on CD25+, FoxP3+, ROR-γt + or T-bet+ was done. CD4 + T cells that expressed one or two transcription factors were evaluated using Boolean combination gates. Representative flow cytometry examples are shown

**Additional file 2: Figure S2.** CD4+ T-cell phenotype is modified by *Mtb*. Recently thawed or freshly isolated PBMCs from HD were stimulated with *Mtb*, stained and analyzed by flow cytometry, as indicated in methods. Figure shows the percentage of CD4 + T cells that express **(A)** FoxP3, T-bet, ROR-γt or CD25 and **(B)** the co-expression of transcription factors using a Boolean gating strategy. **(C)** Tregs (CD4 + CD25 + FoxP3+) and uTregs (CD4 + CD25-FoxP3+) subsets were also analyzed. Representative flow cytometry examples are shown. Each symbol represents an individual subject. Unpaired t test or Mann-Whitney U test, as appropriated *p < 0.05, **p < 0.01 and ***p < 0.005

**Additional file 3: Figure S3.** *Mtb*-specific response in HD is modulated by 7-OD and DHEA. Recently thawed or freshly isolated PBMCs from HD were stimulated with *Mtb* in the presence/absence of 7-OD at 1 × 10^−6^M or DHEA at 1 × 10^−7^M. Then, cells were stained and analyzed by flow cytometry, as described before. Figure shows the percentage of CD4 + T cells expressing **(A)** FoxP3, T-bet, ROR-γt or CD25 and the co-expression of transcription factors using a Boolean gating strategy or **(B)** CD4+ Tregs and uTregs, with the co-expression of the transcription factors ROR-γt or T-bet within each population. Values are relativized to unstimulated cells. The results are plotted for HD. Each symbol represents an individual subject. Friedman test followed by post-hoc comparisons: Fisher’s or Dunn’s test, as appropriated *p < 0.05, **p < 0.01 and ***p < 0.005

**Additional file 4: Figure S4.** The equilibrium between Th1, Tregs, uTregs and Th17 subsets are disrupted by *Mtb* and modified by 7-OD and DHEA. Recently thawed or freshly isolated PBMCs from HD individuals were stained and analyzed by flow cytometry, as indicated in methods. Figure shows CD4 + T cell subset ratios. The results are plotted for HD (open circles) comparing **(A)** Control vs. stimulated cells. Unpaired t test (normal distribution) or Mann-Whitney U test (non-normal variables) *p < 0.05. **(B)** Distinct CD4+ Th subset ratios of *Mtb*-stimulated PBMCs treated with 7-OD and DHEA. Each symbol represents an individual subject. Friedman test followed by post-hoc comparisons: Fisher’s LSD or Dunn’s test, as appropriated *p < 0.05, **p < 0.01 and ***p < 0.005

**Additional file 5: Table S1.** Expression of FoxP3, T-bet, ROR-γt and CD25 on a per cell basis in CD4 + T lymphocytes from HIV-TB patients. Recently thawed or freshly isolated PBMCs from HIV-TB were stimulated with *Mtb* in the presence/absence of 7-OD at 1 × 10^−6^M or DHEA at 1 × 10^−7^M. Then, cells were stained and analyzed by flow cytometry, as described before. Table shows median fluorescence intensity (MFI), which was calculated as the ratio of the geometric mean MFI of the marker of interest over MFI of the corresponding negative population. MFI is expressed as median ± interquartile range (IQR). Friedman test followed by Fisher’s LSD or by Dunn’s test, as appropriate *p < 0.05, **p < 0.01 and ***p < 0.005. * indicates significant differences with *Mtb*-stimulated cells (*Mtb*)

## Data Availability

All data generated or analyzed during this study are included in this published article and its supplementary information files.

## Abbreviations

7-OD: 7-oxo-dehydroepiandrosterone, 7-oxo-DHEA
AET: Androstenetriol
Ag: Antigen
Anti-TB: Antituberculosis treatment
CPM: Counts per minute
DHEA: Dehydroepiandrosterone
HAART: Highly active antiretroviral therapy
HD: Healthy donors
HDT: Host-directed therapies
HIV: Human immunodeficiency virus
HIV-TB: HIV-*Mtb* coinfected patients or HIV-*Mtb* coinfection
IRIS: Immune Reconstitution Inflammatory Syndrome
MFI: Mean fluorescence intensity
*Mtb*: *Mycobacterium tuberculosis*
PBMCs: Peripheral blood mononuclear cells
SEM: Standard error of the mean
TB: Tuberculosis
TF: Transcription factor
Tregs: Conventional regulatory CD4 + T cells (i.e., CD25 + FoxP3)
TST: Tuberculin skin test
uTregs: Unconventional regulatory CD4 + T cells (i.e., CD25-FoxP3+)
