# Supplementary figures and images for: 7-oxo-DHEA enhances impaired M. tuberculosis-specific T cell responses during HIV-TB coinfection
Source: J Biomed Sci. 2020 Jan 6;27:20. doi: 10.1186/s12929-019-0604-z (PMC6943934; doi:10.1186/s12929-019-0604-z)

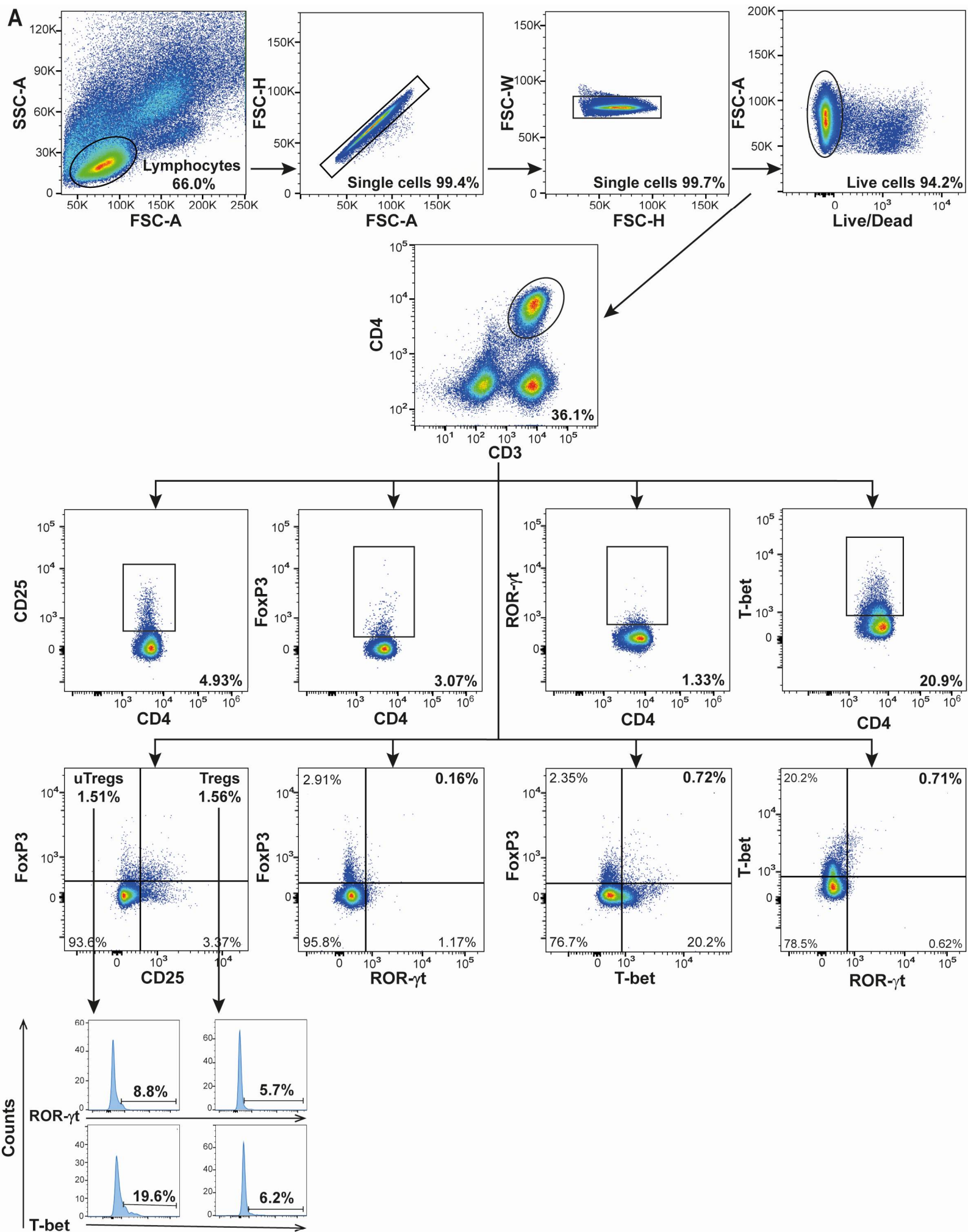

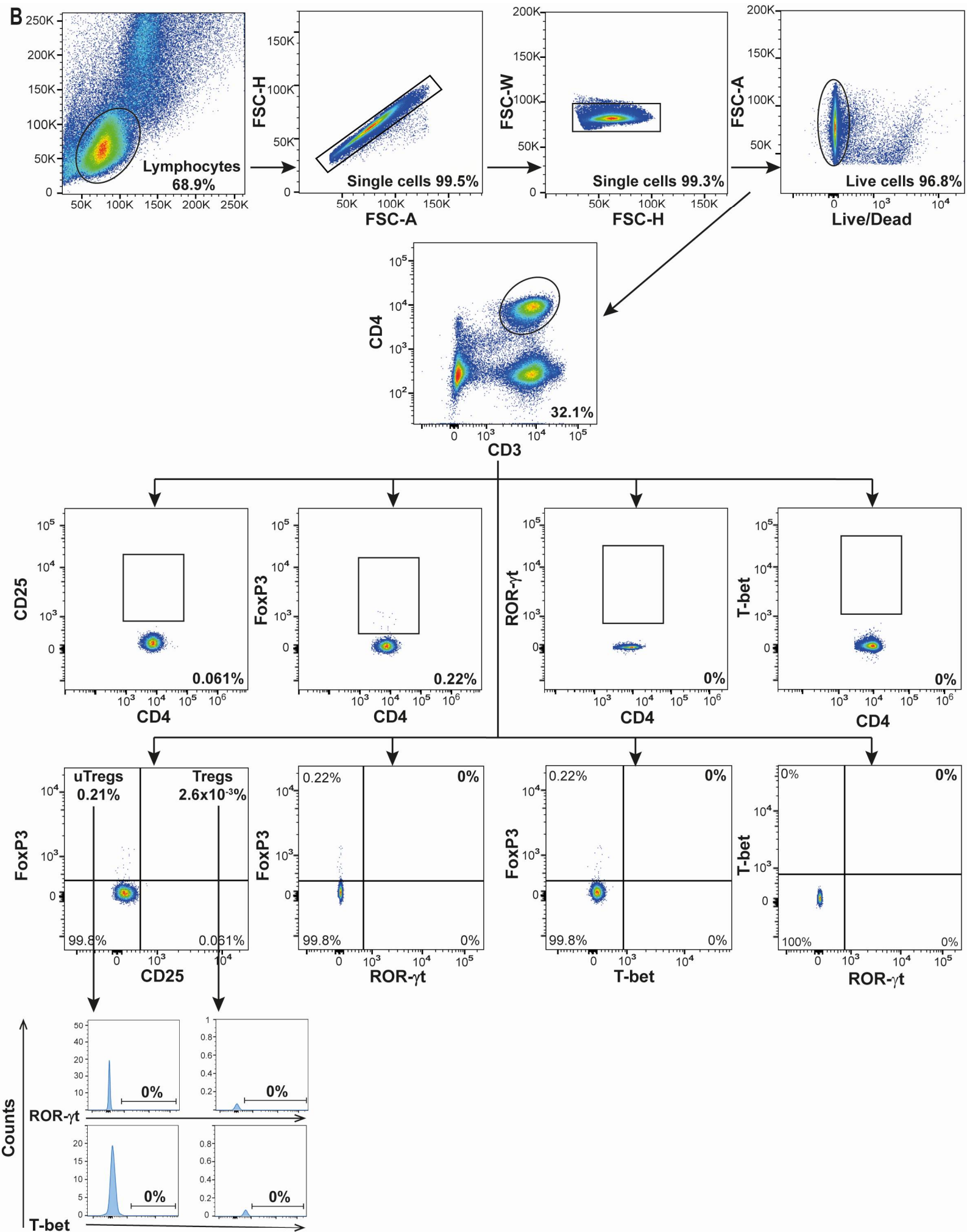

Supplement: Supplementary file 1 — Additional file 1: Figure S1. A representative analysis of the gating strategy developed during this study. Recently thawed or freshly isolated PBMCs were stained and analyzed by flow cytometry. The results are plotted for a sample from a HD labelled with (A) fluorochrome-conjugated Abs or (B) isotype control mAbs. Lymphocyte subset data were generated using a FSC-A/SSC-A gate. After doublet exclusion (FSC-A/FSC-H and FSC-H/FSC-W), live cells were selected using Live/Dead viability probe. Then, CD3 + CD4+ cells were gated and within this group, a gating on CD25+, FoxP3+, ROR-γt + or T-bet+ was done. CD4 + T cells that expressed one or two transcription factors were evaluated using Boolean combination gates. Representative flow cytometry examples are shown [file 12929_2019_604_MOESM1_ESM.pdf]

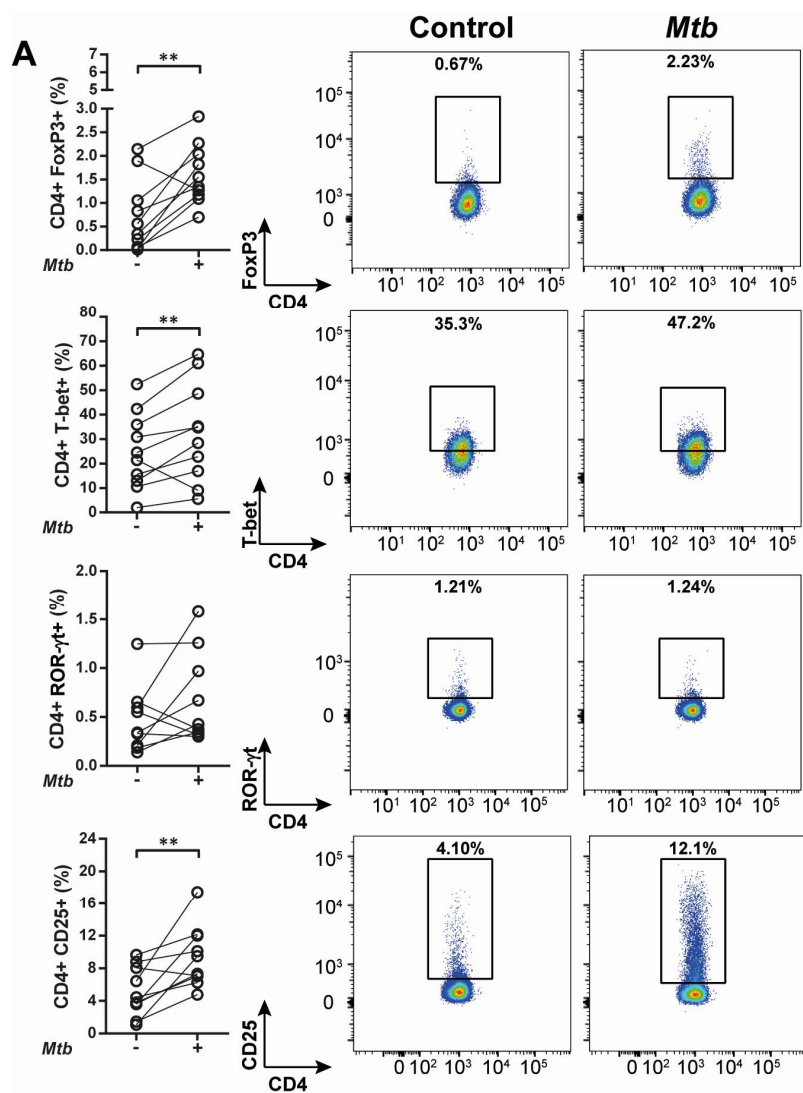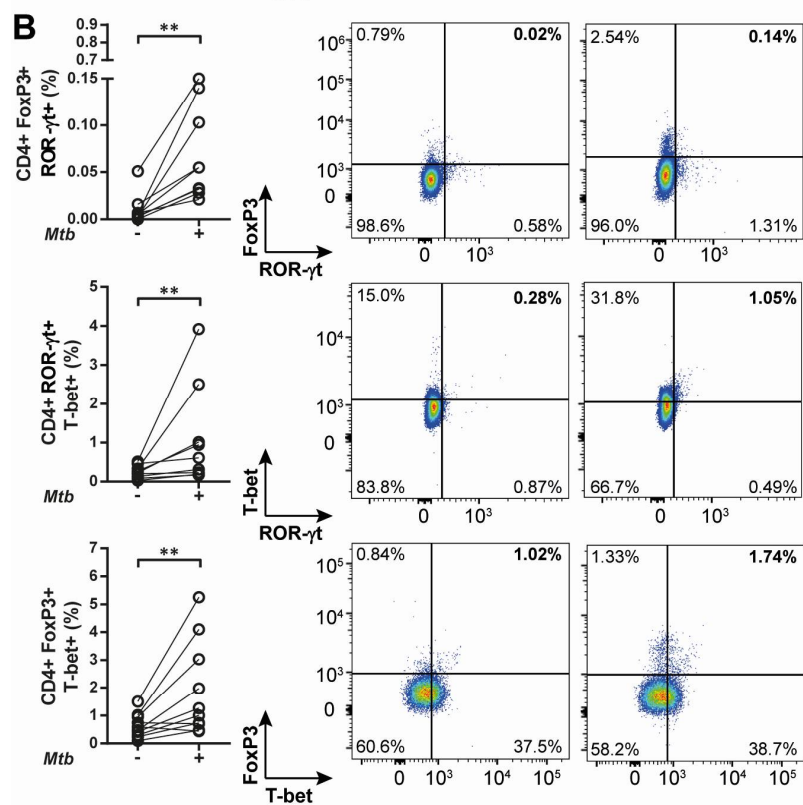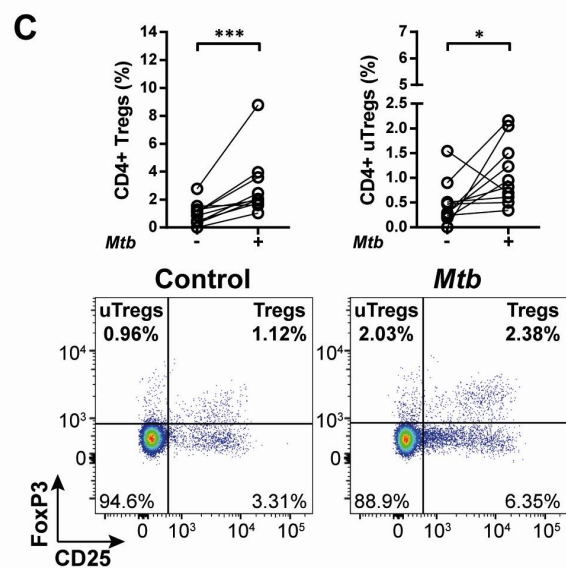

Supplement: Supplementary file 2 — Additional file 2: Figure S2. CD4+ T-cell phenotype is modified by Mtb. Recently thawed or freshly isolated PBMCs from HD were stimulated with Mtb, stained and analyzed by flow cytometry, as indicated in methods. Figure shows the percentage of CD4 + T cells that express (A) FoxP3, T-bet, ROR-γt or CD25 and (B) the co-expression of transcription factors using a Boolean gating strategy. (C) Tregs (CD4 + CD25 + FoxP3+) and uTregs (CD4 + CD25-FoxP3+) subsets were also analyzed. Representative flow cytometry examples are shown. Each symbol represents an individual subject. Unpaired t test or Mann-Whitney U test, as appropriated *p < 0.05, **p < 0.01 and ***p < 0.005 [file 12929_2019_604_MOESM2_ESM.pdf]

**A**

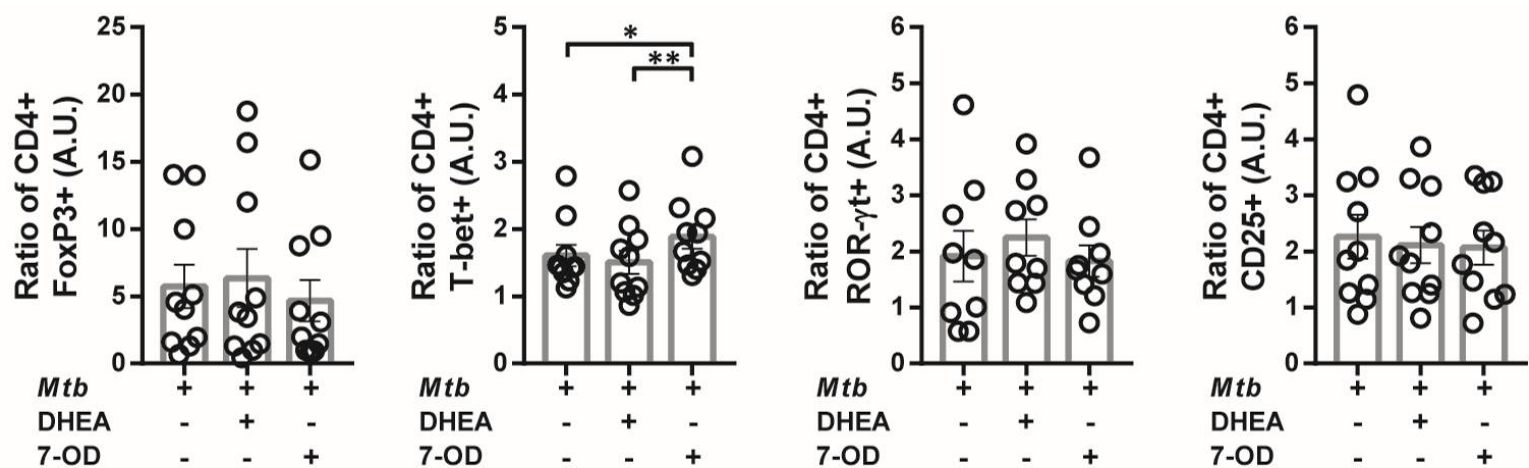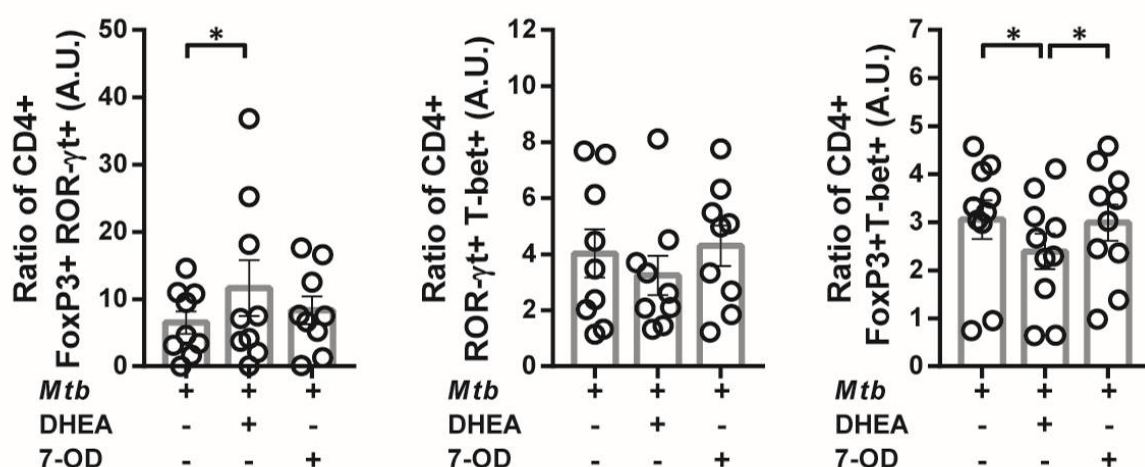

**B**

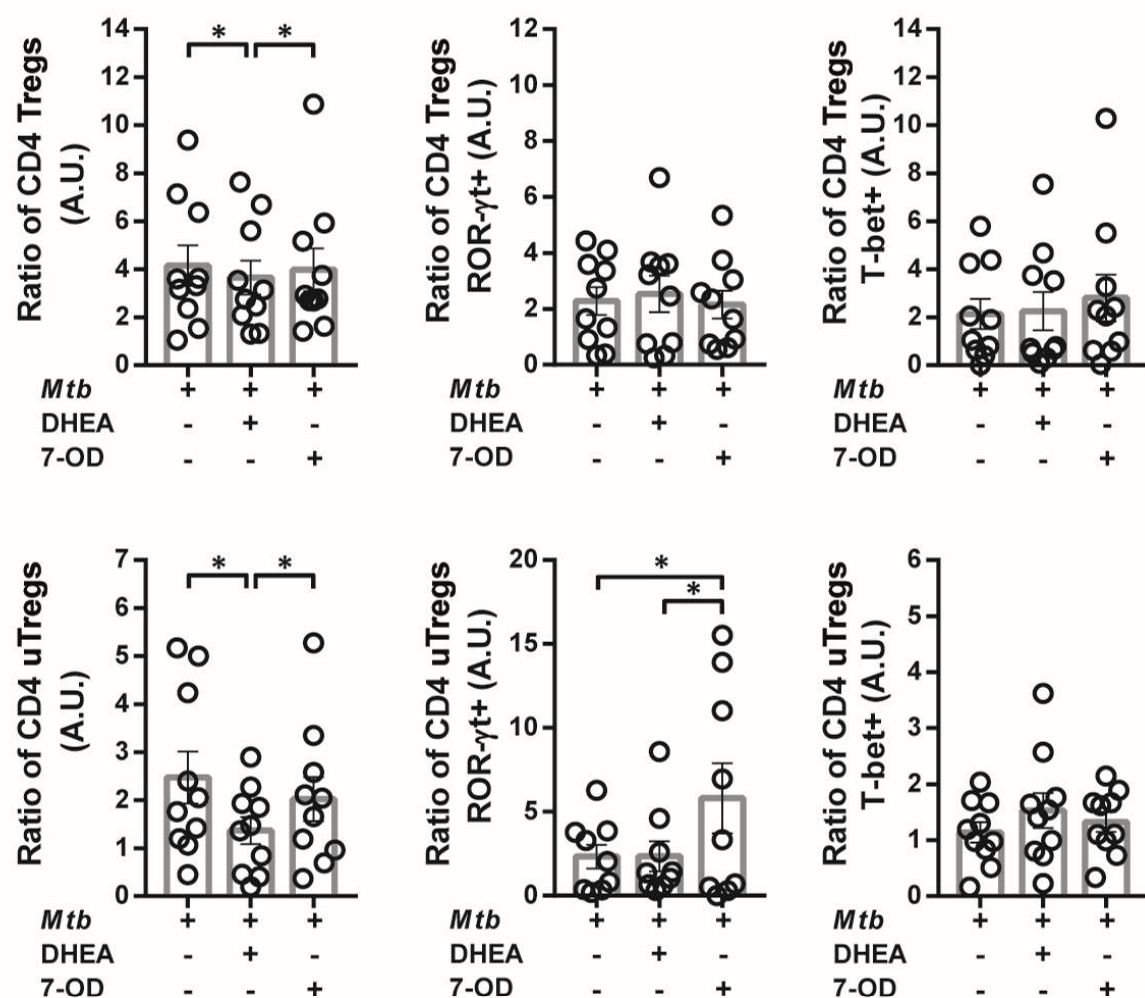

Supplement: Supplementary file 3 — Additional file 3: Figure S3. Mtb-specific response in HD is modulated by 7-OD and DHEA. Recently thawed or freshly isolated PBMCs from HD were stimulated with Mtb in the presence/absence of 7-OD at 1 × 10^−6^M or DHEA at 1 × 10^−7^M. Then, cells were stained and analyzed by flow cytometry, as described before. Figure shows the percentage of CD4 + T cells expressing (A) FoxP3, T-bet, ROR-γt or CD25 and the co-expression of transcription factors using a Boolean gating strategy or (B) CD4+ Tregs and uTregs, with the co-expression of the transcription factors ROR-γt or T-bet within each population. Values are relativized to unstimulated cells. The results are plotted for HD. Each symbol represents an individual subject. Friedman test followed by post-hoc comparisons: Fisher’s or Dunn’s test, as appropriated *p < 0.05, **p < 0.01 and ***p < 0.005 [file 12929_2019_604_MOESM3_ESM.pdf]

**A**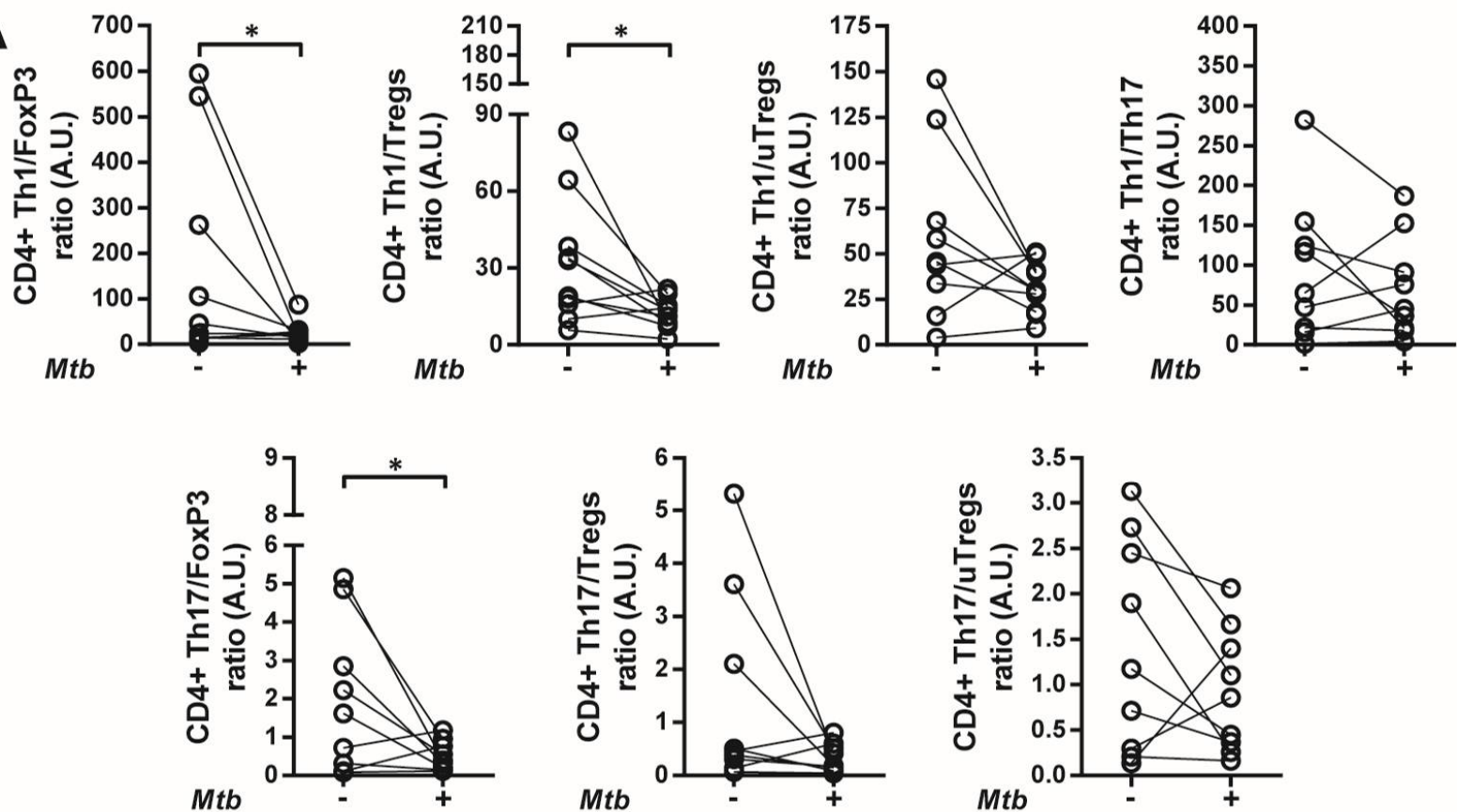**B**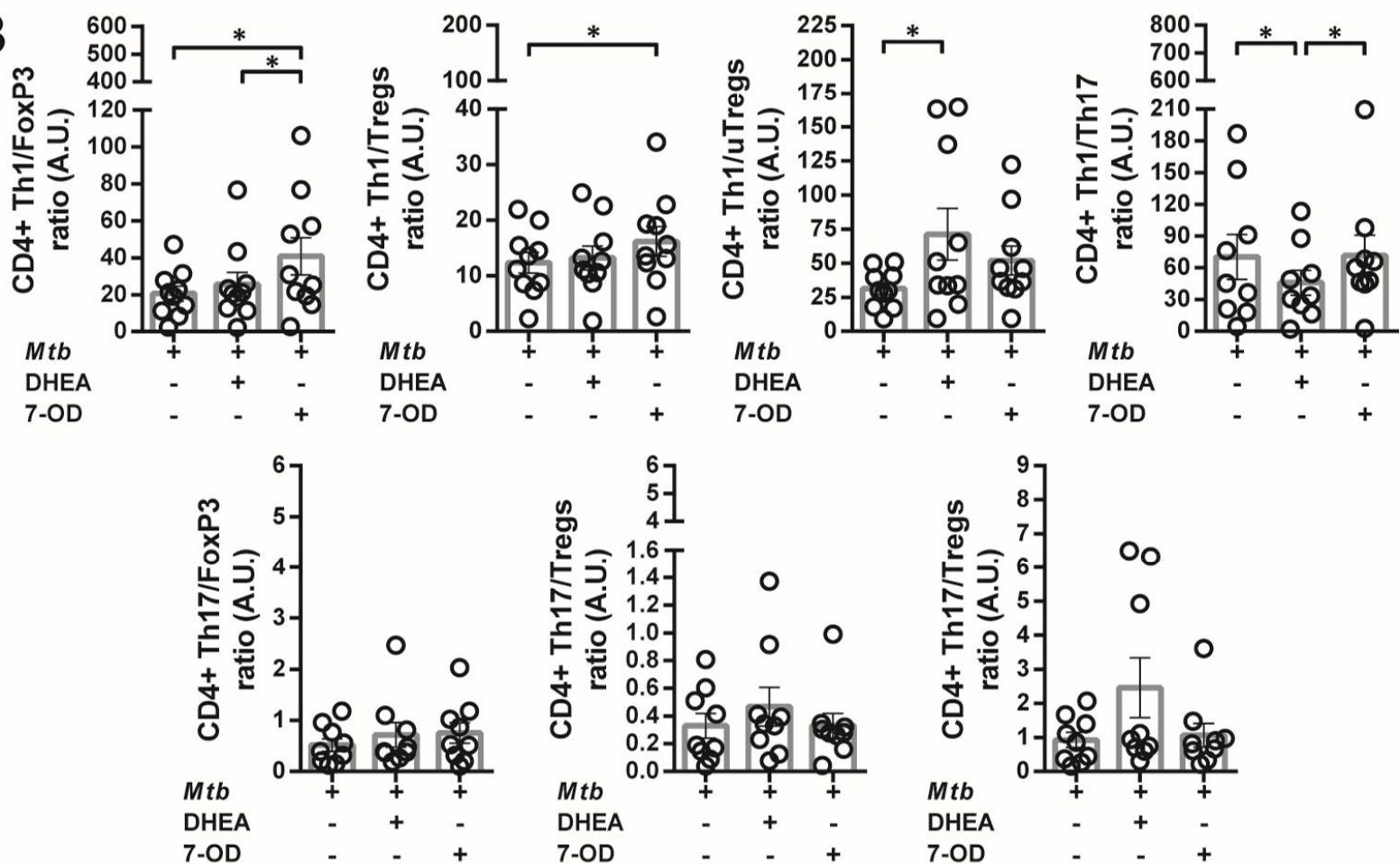

Supplement: Supplementary file 4 — Additional file 4: Figure S4. The equilibrium between Th1, Tregs, uTregs and Th17 subsets are disrupted by Mtb and modified by 7-OD and DHEA. Recently thawed or freshly isolated PBMCs from HD individuals were stained and analyzed by flow cytometry, as indicated in methods. Figure shows CD4 + T cell subset ratios. The results are plotted for HD (open circles) comparing (A) Control vs. stimulated cells. Unpaired t test (normal distribution) or Mann-Whitney U test (non-normal variables) *p < 0.05. (B) Distinct CD4+ Th subset ratios of Mtb-stimulated PBMCs treated with 7-OD and DHEA. Each symbol represents an individual subject. Friedman test followed by post-hoc comparisons: Fisher’s LSD or Dunn’s test, as appropriated *p < 0.05, **p < 0.01 and ***p < 0.005 [file 12929_2019_604_MOESM4_ESM.pdf]
