# Supplementary material for: 7-oxo-DHEA enhances impaired M. tuberculosis-specific T cell responses during HIV-TB coinfection
Source: J Biomed Sci. 2020 Jan 6;27:20. doi: 10.1186/s12929-019-0604-z (PMC6943934; doi:10.1186/s12929-019-0604-z)
Supplement: Supplementary file 5 — Additional file 5: Table S1. Expression of FoxP3, T-bet, ROR-γt and CD25 on a per cell basis in CD4 + T lymphocytes from HIV-TB patients. Recently thawed or freshly isolated PBMCs from HIV-TB were stimulated with Mtb in the presence/absence of 7-OD at 1 × 10^−6^M or DHEA at 1 × 10^−7^M. Then, cells were stained and analyzed by flow cytometry, as described before. Table shows median fluorescence intensity (MFI), which was calculated as the ratio of the geometric mean MFI of the marker of interest over MFI of the corresponding negative population. MFI is expressed as median ± interquartile range (IQR). Friedman test followed by Fisher’s LSD or by Dunn’s test, as appropriate *p < 0.05, **p < 0.01 and ***p < 0.005. * indicates significant differences with Mtb-stimulated cells (Mtb) [file 12929_2019_604_MOESM5_ESM.pdf]

Supplementary Table 1. Expression of FoxP3, T-bet, ROR-γt and CD25 on a per cell basis in CD4+T lymphocytes from HIV-TB patients. Recently thawed or freshly isolated PBMCs from HIV-TB were stimulated with Mtb in the presence/absence of 7-OD at $1x10^{-6}$M or DHEA at $1x10^{-7}$M. Then, cells were stained and analyzed by flow cytometry, as described before. Table shows median fluorescence intensity (MFI), which was calculated as the ratio of the geometric mean MFI of the marker of interest over MFI of the corresponding negative population. MFI is expressed as median ± interquartile range (IQR). Friedman test followed by Fisher's LSD or by Dunn's test, as appropriate *p <0.05, **p <0.01 and ***p <0.005. * indicates significant differences with *Mtb*-stimulated cells (*Mtb*).

	**Unstimulated cells**	***Mtb***	***Mtb*+7OD**	***Mtb*+DHEA**
**FoxP3**	2.22<br>(IQR: 2.05-2.65)	2.31<br>(IQR: 2.15-3.08)	2.72<br>(IQR: 2.29-2.85)	2.33<br>(IQR: 2.17-2.93)
**T-bet**	1.71<br>(IQR: 1.62-1.84)	1.71<br>(IQR: 1.60-1.88)	**1.83 ***<br>**(IQR: 1.67-1.96)**	1.75<br>(IQR: 1.66-1.85)
**ROR-γt**	**2.22 *****<br>**(IQR: 2.00-3.11)**	1.99<br>(IQR: 1.15-2.16)	2.03<br>(IQR: 1.97-2.45)	2.13<br>(IQR: 1.91-2.20)
**CD25**	**3.80 ***<br>**(IQR: 2.76-4.26)**	4.12<br>(IQR: 4.12-5.78)	4.24<br>(IQR: 2.93-5.89)	4.35<br>(IQR: .3.45-5.45)
